# Microstructural White Matter and Links With Subcortical Structures in Chronic Schizophrenia: A Free-Water Imaging Approach

**DOI:** 10.3389/fpsyt.2020.00056

**Published:** 2020-02-27

**Authors:** Tiril P. Gurholt, Unn K. Haukvik, Vera Lonning, Erik G. Jönsson, Ofer Pasternak, Ingrid Agartz

**Affiliations:** ^1^Norwegian Centre for Mental Disorders Research (NORMENT), Division of Mental Health and Addiction, Oslo University Hospital, Oslo, Norway; ^2^Norwegian Centre for Mental Disorders Research (NORMENT), Division of Mental Health and Addiction, Institute of Clinical Medicine, University of Oslo, Oslo, Norway; ^3^Department of Psychiatric Research, Diakonhjemmet Hospital, Oslo, Norway; ^4^Department of Adult Mental Health, Institute of Clinical Medicine, University of Oslo, Oslo, Norway; ^5^Centre for Psychiatry Research, Department of Clinical Neuroscience, Karolinska Institutet, and Stockholm Health Care Services, Stockholm County Council, Stockholm, Sweden; ^6^Psychiatry Neuroimaging Laboratory, Department of Psychiatry, Brigham and Women's Hospital, Harvard Medical School, Boston, MA, United States; ^7^Department of Radiology, Brigham and Women's Hospital, Harvard Medical School, Boston, MA, United States

**Keywords:** psychosis, brain abnormalities, subcortical structures, gray matter, white matter microstructure, free-water imaging, diffusion tensor imaging, magnetic resonance imaging

## Abstract

Schizophrenia is a severe mental disorder with often a chronic course. Neuroimaging studies report brain abnormalities in both white and gray matter structures. However, the relationship between microstructural white matter differences and volumetric subcortical structures is not known. We investigated 30 long-term treated patients with schizophrenia and schizoaffective disorder (mean age 51.1 ± 7.9 years, mean illness duration 27.6 ± 8.0 years) and 42 healthy controls (mean age 54.1 ± 9.1 years) using 3 T diffusion and structural magnetic resonance imaging. The free-water imaging method was used to model the diffusion signal, and subcortical volumes were obtained from FreeSurfer. We applied multiple linear regression to investigate associations between (i) patient status and regional white matter microstructure, (ii) medication dose or clinical symptoms on white matter microstructure in patients, and (iii) for interactions between subcortical volumes and diagnosis on microstructural white matter regions showing significant patient-control differences. The patients had significantly decreased free-water corrected fractional anisotropy (FA_t_), explained by decreased axial diffusivity and increased radial diffusivity (RD_t_) bilaterally in the anterior corona radiata (ACR) and the left anterior limb of the internal capsule (ALIC) compared to controls. In the fornix, the patients had significantly increased RD_t_. In patients, positive symptoms were associated with localized increased free-water and negative symptoms with localized decreased FA_t_ and increased RD_t_. There were significant interactions between patient status and several subcortical structures on white matter microstructure and the free-water compartment for left ACR and fornix, and limited to the free-water compartment for right ACR and left ALIC. The Cohen's d effect sizes were medium to large (0.61 to 1.20, absolute values). The results suggest a specific pattern of frontal white matter axonal degeneration and demyelination and fornix demyelination that is attenuated in the presence of larger structures of the limbic system in patients with chronic schizophrenia and schizoaffective disorder. Findings warrant replication in larger samples.

## Introduction

Schizophrenia is a severe and often debilitating mental disorder with largely unknown disease mechanisms. It is well established that patients with schizophrenia, across different disease states, demonstrate white matter microstructural (1) and gray matter structural (2, 3) differences when compared to healthy controls, as well as progressive differences (4–6) related to the pathophysiology of the disorder and possibly medication use.

Diffusion magnetic resonance imaging (dMRI) and T1-weighted structural imaging are two widely used magnetic resonance imaging (MRI) techniques that are often used to study schizophrenia. dMRI, using its popular analysis method—diffusion tensor imaging (DTI) (7)— yields *in vivo* indirect measures of white matter microstructure (8) such as fractional anisotropy (FA), axial diffusivity (AD) and radial diffusivity (RD). The FA measure can decrease both due to axonal degeneration and demyelination (6, 9), indicated by reduced AD and increased RD, respectively (8). However, the FA measure may not provide a good representation of white matter integrity due to several methodological issues (10), including partial volume effects e.g. from extracellular water contamination and crossing fibers (8). The bi-tensor free-water imaging model (11) accounts for extracellular free-water, yielding improved tissue specificity of white matter measures compared to the DTI model (11). The method also provides a free-water fractional volume measure that is affected by extracellular processes e.g. neuroinflammation, atrophy, and cellular membrane breakdown (12).

The largest DTI meta-analysis to date showed that patients with schizophrenia have widespread white matter FA reductions compared to controls, with regionally more severe differences with increasing illness duration (1). Cross-sectional free-water imaging studies in schizophrenia corroborate increasing tissue change with illness duration; At schizophrenia onset, reports indicate limited tissue change together with widespread increase in free-water (13, 14), while with chronicity there is evidence of widespread tissue changes together with limited free-water increase (12, 15) when compared to healthy controls. These findings could indicate a severity gradient and that the temporal disease state needs to be considered in schizophrenia studies of microstructural white matter.

Cross-sectional structural MRI studies have shown alterations of subcortical volumes, including smaller hippocampus and amygdala, and larger basal ganglia volumes in schizophrenia patients when compared to healthy controls (2, 16, 17). Further, enlargement of the putamen and pallidum volumes with age and illness duration has been reported (2). Studies also report cortical thinning in patients compared to controls (3, 16, 18, 19), which has been linked to microstructural white matter alterations in patients with schizophrenia as indicated by reduced FA (20, 21). Recently cortical thinning was also inversely associated with infracortical white matter anisotropy in adult patients (< 50 years of age) (22). This could indicate patterns of associations between brain regions that are limited to patients with schizophrenia. Although a single study have shown increased mean diffusivity of the left accumbens, and the hippocampus and thalamus bilaterally, in patients (23), the putative link between white matter microstructure and subcortical structures remains understudied.

In the present study we investigated white matter diffusion properties using the free-water imaging method. Based on prior free-water imaging studies, we expected microstructural white matter alterations in patients with chronic schizophrenia and schizoaffective disorder together with limited evidence of increased free-water (12, 15), and clinical symptoms to be linked with microstructural white matter in patients (15). Further, based on prior studies using the standard DTI method, we hypothesized that white matter microstructure could be associated with medication use (24, 25), and that patient white matter microstructure could be differently associated with volumetric subcortical measures than in controls, similar to prior cortical findings (20, 21). The aims of this study were to (i) identify differences in microstructural white matter diffusion properties between long-term treated patients with schizophrenia and schizoaffective disorder, and healthy controls, (ii) investigate putative associations between medication or clinical symptoms on white matter microstructure in patients, and (iii) investigate whether volumetric measures of subcortical brain structures were associated with observed patient-control differences in white matter microstructure.

## Materials and Methods

### Study Population

The subject sample consisted of 30 patients [schizophrenia (n = 22), schizoaffective disorder (n = 8)] and 42 controls, recruited among participants from the Human Brain Informatics Project (HUBIN) study at the Karolinska Hospital (18, 26), and investigated between 2011 and 2015. Exclusion criteria for all participants were age <18 or >70 years, IQ < 70, or previous severe head injury. All participants received oral and written information about the study and signed a written informed consent. The study was approved by the Regional Ethical Review Board of Stockholm, Sweden (Dnr 2009/1465-31/3), and was conducted in accordance with the Helsinki declaration.

### Clinical Assessment

Patients and controls were assessed by a psychiatrist (EGJ) using the Structured Clinical Interview for DSM-III-R axis I disorders (27). Diagnosis was based on DSM-IV (28). Symptoms were assessed according to the Scale for the Assessment of Negative Symptoms (SANS) (29) and the Scale for the Assessment of Positive Symptoms (SAPS) (30). Psychosocial functioning was assessed using the split version of the Global Assessment of Function (GAF-S and GAF-F) scale (31). Age at onset was defined as the age of first verified positive psychotic symptom experience and duration of illness was calculated in years from age at onset to age at MRI. Chlorpromazine equivalent antipsychotic dose (CPZ) was computed (32).

### Data Acquisition

Patients and controls underwent MRI on the same 3 T General Electric Healthcare Discovery MR750 Sigma scanner (General Electric Company, Milwaukee, Wisconsin, USA) equipped with an 8-channel head coil at the Karolinska Institutet and Hospital. Axial diffusion MRI data were acquired with anterior-to-posterior phase-encoding direction, 10 b_0_ volumes and 60 diffusion weighted volumes with b = 1,000 s/mm^2^. The scanning parameters were: 128 × 128 acquisition matrix, repetition time (TR) = 6.0 s, echo time (TE) = 82.9 ms, field of view = 240 mm, flip angle = 90° and spatial resolution 0.94 × 0.94 × 2.9 mm^3^. A sagittal T1-weighted BRAVO sequence was acquired with TR = 7.9 s, TE = 3.06 s, inversion time (TI) = 450 ms, flip angle = 12°, field of view = 240 mm and voxel size = 0.94 × 0.94 × 1.2 mm^3^. There was no major scanner upgrade or change of instrument during the study period.

### MRI Processing

All dMRI's were processed as follows: Brain masks of the first b_0_ volume were manually edited to remove non-brain tissue. The dMRI's were corrected for eddy current induced distortions and subject head motion using FSLs EDDY (33). We enabled automatic detection and correction of motion induced signal dropout (34), previously shown to enhance signal-to-noise ratio (35). EDDY outputs rotated b-vectors used in subsequent processing and total per-volume-movement used to calculate the average motion. Following EDDY correction, a bi-tensor diffusion model was fitted using a nonlinear regularized fit to obtain a free-water corrected diffusion tensor representing the tissue compartment and the fractional volume of an isotropic free-water compartment (11). From the diffusion tensor, a tissue specific scalar measurement of fractional anisotropy (FA_t_) was derived using FSLs dtifit. FA_t_ depends on two independent measures, radial diffusivity (RD_t_) and axial diffusivity (AD_t_), and they were derived for an additional level of investigation. The scalar measurements of each subject were projected onto a standard FA skeleton using Tract-Based Spatial Statistics (TBSS) (36). To do so, the FA images were registered to the ENIGMA-DTI FA template (37) that aligns with the Johns Hopkins University DTI atlas (38) following the ENIGMA-DTI processing protocols (http://enigma.ini.usc.edu/protocols/dti-protocols/). Forty-four regions of interests (ROIs; **Table 1**) were extracted. Further, we derived the standard DTI measures of AD, RD, and mean diffusivity (MD) and projected them onto the FA skeleton for an extra level of investigation, as recommended in a recent review (39).

**Table 1:** Overview of the investigated white matter regions of interest.

Abbreviation	Full name
ACR*	Anterior corona radiata
ALIC*	Anterior limb of internal capsule
*Average*	Average of diffusion measure
*BCC*	Body of corpus callosum
*CC*	Corpus callosum
CGC*	Cingulum
CGH*	Cingulum (hippocampal portion)
CR*	Corona Radiata
CST*	Corticospinal tract
EC*	External capsule
*FX*	Fornix
FXST*	Fornix stria terminalis
*GCC*	Genu of corpus callosum
IC*	Internal capsule
IFO*	Inferior fronto occipital fasciculus
PCR*	Posterior corona radiata
PLIC*	Posterior limb of internal capsule
PTR*	Posterior thalamic radiation
RLIC*	Retrolenticular part of IC
*SCC*	Splenium of corpus callosum
SCR*	Superior corona radiata
SFO*	Superior fronto-occipital fasciculus
SLF*	Superior longitudinal fasciculus
SS*	Sagittal stratum
UNC*	Uncinate

*Bilateral structures.

All T1-weighted MRI scans were processed using FreeSurfer (40) version 6.0.0. The processing steps include motion correction, bias field correction, brain extraction, intensity normalization and automatic Talairach transformation, with optimized 3 T bias field filtration (41). Subcortical volumes were obtained through the subcortical segmentation stream (42), except for the hippocampus and amygdala structures obtained through joint segmentation in FreeSurfer version 6.0.0, development version (43, 44). The extracted subcortical structures were: hippocampus, amygdala, thalamus, nucleus accumbens, caudate, pallidum, putamen and lateral ventricle.

### MRI Quality Control

Only dMRI's and volumetric structures passing quality control were included in the analyses. Initially there were 80 participants in the study.

All DWIs were visually inspected in three orthogonal views for severe visible artifacts (45), leading to the exclusion of 4 participants. Additionally, we excluded 4 subjects with an EDDY estimated average motion above two standard deviations from the mean. After quality control there were 72 participants in the study.

All T1-weighted images were visually inspected for movement and cortical parcellation errors. No participants needed to be excluded at this stage. The segmentation quality of the subcortical volumes was assessed by manual inspection of outlier volumes (defined as: ≥1.5 times interquartile range). Outlier volumes were excluded if the segmentation was inaccurate. This led to the exclusion volumes from four participants, namely: one volume each for the left amygdala, left thalamus, left putamen, right putamen, right accumbens, and right caudate.

### Statistical Method

The demographic variables of patients and controls were compared using χ^2^-test for categorical variables, two-sample t-test/two-sided Wilcoxon rank-sum test for normally/non-normally distributed continuous variables. Normality was assessed using the Shapiro–Wilk's normality test (46).

In the main analyses, we applied multiple linear regression to assess the effect of patient-control differences on each white matter ROIs using the *lm*-function in R (version 3.5.0). For comparison, we included analyses using both the free-water imaging and standard DTI method. In patients, we further investigated free-water imaging metrics for the effects of medication and clinical symptoms on each ROI using similar models. For all models we adjusted for sex, age, and average movement.

We conducted follow-up analyses for ROIs showing significant patient-control differences to assess potential associations between white matter microstructure and volumetric measures of subcortical structures. To do so, we extended the main model to include a term for the subcortical volumes and its interaction with patient status. We did not adjust for the intracranial volume since we investigate diffusion properties of white matter microstructure as the dependent variable, and we wanted to capture associations between diffusion properties and subcortical volumes, without also adjusting for the intracranial volume.

We computed Cohen's d effect size from the t-statistics for categorical variables, and *via* the partial correlation coefficient, r, for continuous variables (47). We corrected for multiple comparisons using the false discovery rate (FDR) at α = 0.05 (48) across planned analyses, yielding significance threshold p ≤ 0.0109. For follow-up analyses, a separate FDR threshold was computed at p ≤ 0.0116.

## Results

### Demographic and Clinical Data

Patients had an average age at onset of 23.5 ± 4.6 years and an average duration of illness of 27.6 ± 8.0 years. Of the patients, 93.3% received antipsychotic medication (8 first generation, 13 second generation, 7 first and second generation) with an average CPZ dose of 409.8 ± 325.2 mg. Compared to the controls, patients had significantly fewer years of education (p = 0.0011), and decreased functioning as assessed by GAF symptom (p = 6.2e-13) and GAF function (p = 1.4e-13) score. During diffusion MRI, the patients moved significantly more than the controls (p = 0.0298). There were no significant differences in the other clinical or demographic data (**Table 2**).

**Table 2:** Demographics and clinical variables.

Clinical information	Patients (n = 30)	Healthy Controls (n = 42)	χ^2^-test/Wilcoxon rank sum test/t-test	p-value
*Women, N (%)*	8 (26.7)	13 (30.9)	0.02	0.8954
*Age (years)*	51.1 ± 7.9	54.1 ± 9.1	-1.43	0.1581
*Education (years)*	12.9 ± 2.2	15.1 ± 3.1	-3.40	**0.0011**
*Handedness (R/L/A)*^a^	21/4/2	37/3/1	2.10	0.3506
*AAO (years)*	23.5 ± 4.6			
*DOI at MRI (years)*	27.6 ± 8.0			
*AP Medicated, N (%)*	28 (93.3)			
*FGA/SGA/mixed, N*	8/13/7			
*CPZ (mg)*	409.8 ± 325.2			
**Clinical measurements**				
*SAPS total*	9.2 ± 7.9			
*SANS total*	27.1 ± 13.8			
*GAF-S^b^*	46.5 ± 10.0	81.7 ± 9.0	-35.00	**6.2e-13**
*GAF-F^b^*	45.5 ± 8.9	86.7 ± 7.6	-40.00	**1.4e-13**

Significance threshold p < 0.05 indicated in bold. Two-sample t-test/two-sided Wilcoxon rank-sum test applied for normally/non-normally distributed continuous data. χ^2^-test applied for categorical data. A, Ambidextrous; AAO, age at onset; AP, antipsychotic medication; CPZ, chlorpromazine equivalent antipsychotic dose; DOI, duration of illness; FGA, first generation antipsychotics; GAF, Global Assessment of Functioning; GAF-F, GAF function scale; GAF-S, GAF symptom scale; L, Left; MRI, magnetic resonance imaging; Patients, schizophrenia patients; R, Right; SAPS, Scale for the Assessment of Positive Symptoms; SANS, Scale for the Assessment of Negative Symptoms; SGA, second generation antipsychotics.

a Three patients and one control had missing data on handedness.

b Data not normally distributed.

### Patient–Control Differences in Diffusion Properties

**Figure 1:**
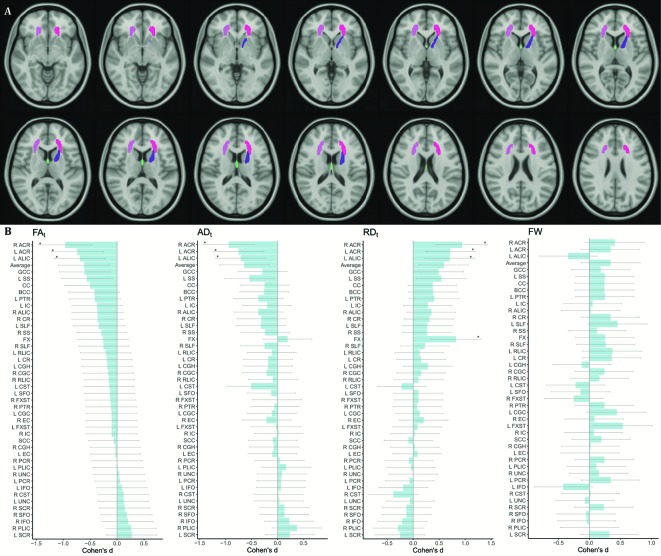
**(A)** shows ROIs with significant patient-control differences are illustrated on the MNI 152 T1 atlas. **(B)** shows Cohen**'**s d effect sizes for each ROI and free-water diffusion metric of patient-control differences, ordered by ascending effect sizes for FA_t_. ROIs that pass the FDR threshold of p ≤ 0.0109 are indicated with *. ACR, anterior corona radiata; AD_t_, FW adjusted axial diffusivity; ALIC, anterior limb of internal capsule; Average, average of diffusion metric; BCC, body of corpus callosum; CC, corpus callosum; CGC, cingulum; CGH, cingulum hippocampal portion; CR, corona radiata; CST, corticospinal tract; EC, external capsule; FA_t_, FW adjusted fractional anisotropy; FW, free-water; FX, fornix; FXST, fornix stria terminalis; GCC, genu of corpus callosum; IC, internal capsule; IFO, inferior fronto occipital fasciculus; L, Left; PCR, posterior corona radiata; PLIC, posterior limb of internal capsule; PTR, posterior thalamic radiation; R, Right; RD_t_, FW adjusted Radial diffusivity; RLIC, retrolenticular part of IC; ROI, region of interest; SCC, splenium of corpus callosum; SCR, superior corona radiata; SFO, superior fronto-occipital fasciculus; SLF, superior longitudinal fasciculus; SS, sagittal stratum; UNC, uncinate.

FA_t_ was significantly lower in patients compared to controls in the right anterior corona radiata (ACR) (d = -0.96, p = 0.0002), left ACR (d = -0.74, p = 0.0040) and left anterior limb of internal capsule (ALIC) (d = -0.69, p = 0.0069) (**Figure 1**; **Table S1**). In those regions, FA_t_ reductions were driven by significantly lower AD_t_ (right ACR: d = -0.94, p = 0.0003; left ACR: d = -0.75, p = 0.0035; and left ALIC: d = -0.71, p = 0.0058) and significantly higher RD_t_ (right ACR: d = 0.94, p = 0.0003; left ACR: d = 0.71, p = 0.0055; and left ALIC: d = 0.70, p = 0064). Furthermore, the RD_t_ of the fornix was significantly higher for patients (d = 0.82, p = 0.0015) without any corresponding significant or nominal-significant differences in FA_t_ or AD_t_. There were nonsignificant patient-control differences in free-water.

For comparison, running the same analysis using the standard DTI model showed significant differences between patients and controls only for the right ACR (FA: d = -0.88, p = 0.0007; RD: d = 0.66, p = 0.0100) (**Table S2**).

### Effects of Medication

Analyses in patients did not show any significant CPZ medication effects on white matter microstructure (**Table S3**).

### Effects of Clinical Symptoms

Analyses in patients showed that total SAPS scores were significantly associated with increased free-water on the right posterior thalamic radiation (PTR: d = 1.20, p = 0.0061) and the left sagittal stratum (SS: d = 1.16, p = 0.0078), respectively (**Table S4**). Total SANS scores were for the right ALIC significantly associated with decreased FA_t_ (d = -1.14, p = 0.0088) and increased RD_t_ (d = 1.20, p = 0.0061) (**Table S5**).

### Association Between Diffusion Measures and Subcortical Volumes

In ROIs with significant diagnostic differences, we conducted follow-up analyses for association between free-water imaging diffusion metrics and subcortical volumes identified as significant interaction between subcortical volumes and patient status.

**Figure 2:**
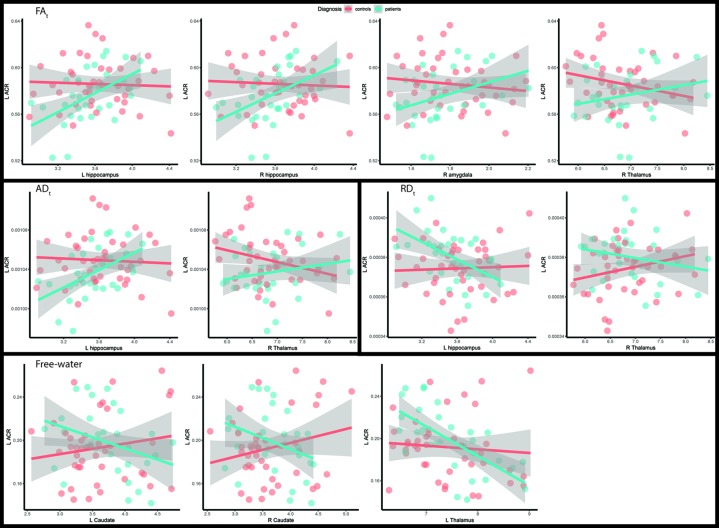
Scatterplots of the left anterior corona radiata with the subcortical structures that have significant interaction with diagnosis. Fitted lines were created using a generalized additive model. AD_t_, Free-water adjusted axial diffusivity; ACR, anterior corona radiata; FA_t_, Free-water adjusted fractional anisotropy; L, Left; RD_t_, Free-water adjusted radial diffusivity; R, Right.

For the left ACR, the FA_t_ reduction in patients was attenuated in the presence of larger measures of hippocampus (left: d = 0.77, p = 0.0028; right: d = 0.69, p = 0.0068), right amygdala (d = 0.67, p = 0.0084), and right thalamus (d = 0.68, p = 0.0076). Similarly, AD_t_ reduction and RD_t_ increase were attenuated for larger left hippocampus (AD_t_: d = 0.72, p = 0.0048; RD_t_: d = -0.7, p = 0.0064) and right thalamus (AD_t_: d = 0.67, p = 0.0084; RD_t_: d = -0.66, p = 0.0098). For the free-water compartment, despite non-significant main effect of diagnosis, free-water was attenuated in the presence of larger caudate (left: d = -0.69, p = 0.0069; right: d = -0.71, p = 0.0057) and left thalamus (d = -0.69, p = 0.0075) (**Figure 2**; **Table S6**).

**Figure 3:**
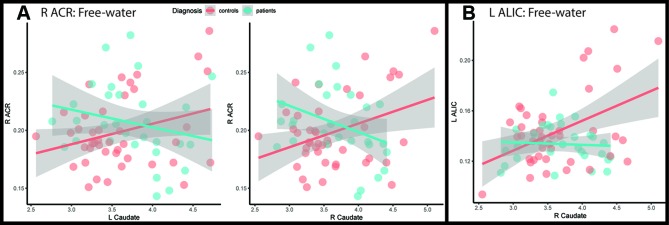
Scatterplots of the **(A)** right anterior corona radiata and **(B)** left anterior limb of internal capsule with the subcortical structures that have significant interaction with diagnosis. Fitted lines were created using a generalized additive model. ACR, anterior corona radiata; ALIC, anterior limb of internal capsule; L, Left; R, Right.

For the right ACR and left ALIC, despite non-significant main effect of diagnosis, we found associations between free-water and caudate in patients; the free-water compartment was reduced for the right ACR in the presence of larger caudate (left: d = -0.67, p = 0.0091; right: d = -0.85, p = 0.0012), and for the left ALIC for larger right caudate (d = -0.65, p = 0.0111) (**Figure 3**; **Table S7** and **S8**).

**Figure 4:**
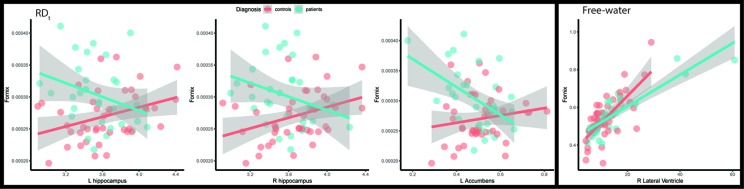
Scatterplots of the fornix with the subcortical structures that have significant interaction with diagnosis. Fitted lines were created using a generalized additive model. L, Left; RD_t_, Free-water adjusted Radial diffusivity; R, Right.

For the fornix, RD_t_ was reduced for larger left nucleus accumbens (d = -0.73, p = 0.0044) and hippocampus (left: d = -0.68, p = 0.0083; right: d = -0.66, p = 0.0100). The free-water compartment was, despite non-significant main effect of diagnosis, significantly associated with right ventricle (d = -0.69, p = 0.0071) in patients (**Figure 4**; **Table S9**).

## Discussion

In this study we investigated the relationship between free-water imaging measures and subcortical volumes in patients with long-term treated chronic schizophrenia and schizoaffective disorder. The main findings were localized lower white matter FA_t_ in patients compared with healthy controls, but no differences in free-water. White matter microstructure was linked with subcortical volumes in differing patterns for patients and controls.

The observed reduction in FA_t_, driven by a combination of AD_t_ reduction and RD_t_ increase, could indicate a pattern of axonal degeneration and demyelination in the frontal white matter in long-term treated patients with chronic schizophrenia and schizoaffective disorder when compared to controls. In the fornix, the increased RD_t_ without simultaneous reduced FA_t_ and AD_t_ could imply demyelination without axonal degeneration in the patients. The free-water imaging method was more sensitive to patient-control differences in white matter microstructure than the standard DTI model. Our findings were similar to and partly overlapping with previous free-water imaging studies indicating reduced FA_t_ in cross-hemisphere frontal white matter in patients with schizophrenia (12–15), and no free-water increase in the chronic state (12, 15). The FA_t_ changes were more pronounced than the reported changes in first episode patients (13, 14). However, the FA_t_ changes were not as widespread as the previously reported changes in chronic schizophrenia (12, 15). The differences could be due to the limited sample size in the current study, or the inclusion of patients diagnosed with schizoaffective disorder, but could also reflect that our long-term treated patient sample on average had been ill for 27 years. This is longer than the previous studies, and it is not known how the disorder progress with age and illness duration as captured by dMRI and compared to healthy controls.

The observed interactions between patient status and subcortical structures on white matter microstructure for both the left ACR and the fornix, indicate patterns of association between the structures that are different in patients with chronic schizophrenia and schizoaffective disorder compared to healthy controls. This is in line with previous studies that show cortical thinning in relation to white matter changes in patients with schizophrenia (20–22). The results may suggest that volumetric properties of brain anatomical structures are related to disrupted white matter microstructure in schizophrenia and schizoaffective disorder. The findings were in the direction of larger subcortical structures being associated with less severe white matter changes in patients with chronic schizophrenia and schizoaffective disorder, or vice versa. This could point towards a severity gradient in structural changes where less pronounced microstructural changes in patients have weaker diagnosis specific links to subcortical structures. They could also indicate disease specific patterns of associations between subcortical structures and microstructural white matter properties in chronic schizophrenia and schizoaffective disorder, and of disrupted functioning of the limbic system (49) and prefrontal connections (50). The reported findings are in line with the hypothesis of schizophrenia being a disorder of dysconnectivity (51–53). It is well established that the limbic system plays a role in schizophrenia, and subcortical structures of the limbic system have previously been reported as reduced in schizophrenia (2). The current study provides further support for limbic system involvement in the disorder together with links to white matter structures.

We did not find any general patient-control differences in free-water. Despite this, in the follow-up analyses we found evidence of diagnosis specific involvement between free-water and some subcortical structures, and particularly larger caudate. Thus, there could be diagnosis specific association patterns between free-water and subcortical structures, but the implication of this is unknown. We can only speculate that the associations could be linked to e.g. better functioning or reduced inflammatory state.

Among patients, positive symptoms were significantly associated with increased free-water in the right PTR and left SS. Similarly, negative symptoms were associated with reduced FA_t_ and increased RD_t_ in the right ALIC. This is in line with a prior study indicating that positive symptoms are associated with increased free-water and negative symptoms with reduced FA_t_ (15). Furthermore, negative and positive symptoms were recently linked to changes in white matter microstructure using standard DTI (1). The association between microstructural white matter and clinical symptoms needs further investigation.

We did not observe any CPZ medication effects on white matter microstructure, which is in line with prior research (1). However, given the long-term treatment of the patients in the current study we cannot rule out that the observed effects on brain structure are due to antipsychotic medication, although here not captured by CPZ. First and second generation antipsychotic medication could be differently involved with brain microstructure as previously shown for basal ganglia structures (54), but this could not be addressed in the current study.

This study had some limitations. The cross-sectional design makes it difficult to distinguish cause from effect. We did not adjust for handedness which could be associated with laterality differences. Moreover, although the effect sizes were strong, the results were only partially overlapping with prior free-water imaging studies in chronic schizophrenia (12, 15). The limited sample size implies higher uncertainty in the effect size estimate (55), and the results need replication in larger independent samples. Further, we limited our study sample to schizophrenia and schizoaffective disorder, and did not investigate psychosis across the broader psychosis spectrum. Strengths of the study includes a well characterized patient sample that has been characterized with research assessment by one psychiatrist for 12 years, 3 T dMRI acquisition of good quality, detailed description of analysis pipeline, validated and robust analysis methods, FDR correction for multiple comparison, and medium to strong effect sizes.

*To conclude*, this study provides further evidence for white matter abnormalities, as well as evidence for altered involvement of subcortical structures with white matter microstructure, in patients with chronic schizophrenia and schizoaffective disorder when compared to healthy controls. The microstructural white matter differences indicate a process of frontal axonal degeneration and demyelination, and fornix demyelination in the patients. Positive and negative psychosis symptoms were associated with free-water and microstructural tissue properties, respectively. The observed interaction between subcortical structures and patient status on white matter microstructure could indicate disease specific patterns of associations between the structures, limited to patients. To fully capture the linkage between gray and white matter tissue in chronic schizophrenia and schizoaffective disorder, future studies in larger samples are needed.

## Data Availability Statement

The datasets from this study will not be made publicly available as we do not have approvals for sharing clinical data.

## Ethics Statement

The study was approved by the Regional Ethical Review Board of Stockholm, Sweden (Dnr 2009/1465-31/3). The patients/participants provided their written informed consent to participate in this study.

## Author Contributions

TPG designed the study in collaboration with OP and IA. TPG did the literature search and drafted the manuscript and interpreted the data together with OP and UKH. VL contributed with clinical insight and data interpretation. OP contributed with the free-water imaging method. EGJ and IA obtained funding, acquired the data, and contributed to study design. TPG, UKH, EGJ, OP, and IA contributed to the final manuscript. All authors approved the final manuscript.

## Funding

This work was funded by The Swedish Research Council (K2012-61X-15078-09-3, K2015-62X-15077-12-3, and 2017-00949), the regional agreement on medical training and clinical research between Stockholm County Council and the Karolinska Institutet; The Research Council of Norway (#223273); KG Jebsen Foundation (#SKGJ-MED-008); South-Eastern Norway Regional Health Authority (#2017112); and the National Institutes of Health (R01MH108574).

## Conflict of Interest

The authors declare that the research was conducted in the absence of any commercial or financial relationships that could be construed as a potential conflict of interest.

## References

[1] Kelly S Jahanshad N Zalesky A Kochunov P Agartz I Alloza C Widespread white matter microstructural differences in schizophrenia across 4322 individuals: results from the ENIGMA Schizophrenia DTI Working Group. Mol Psychiatry (2017) 23: 1261–9. 10.1038/mp.2017.170. PMC5984078 29038599

[2] van Erp TG Hibar DP Rasmussen JM Glahn DC Pearlson GD Andreassen OA Subcortical brain volume abnormalities in 2,028 individuals with schizophrenia and 2,540 healthy controls *via the* ENIGMA consortium. Mol Psychiatry (2016) 21:585. 10.1038/mp.2015.118 26283641 PMC5751698

[3] van Erp TGM Walton E Hibar DP Schmaal L Jiang W Glahn DC Cortical brain abnormalities in 4474 individuals with schizophrenia and 5098 control subjects *via the* enhancing neuro imaging genetics through meta analysis (ENIGMA) consortium. Biol Psychiatry (2018) 84: 644–54. 10.1016/j.biopsych.2018.04.023 PMC6177304 29960671

[4] Dietsche B Kircher T Falkenberg I Structural brain changes in schizophrenia at different stages of the illness: A selective review of longitudinal magnetic resonance imaging studies. Aust N Z J Psychiatry (2017) 51:500–8. 10.1177/0004867417699473 28415873

[5] Kochunov P Hong LE Neurodevelopmental and neurodegenerative models of schizophrenia: white matter at the center stage. Schizophr Bull (2014) 40:721–8. 10.1093/schbul/sbu070 PMC4059450 24870447

[6] Samartzis L Dima D Fusar-Poli P Kyriakopoulos M White matter alterations in early stages of schizophrenia: a systematic review of diffusion tensor imaging studies. J Neuroimaging (2014) 24:101–10. 10.1111/j.1552-6569.2012.00779.x 23317110

[7] Basser PJ Mattiello J LeBihan D diffusion tensor spectroscopy MR and imaging. Biophys J (1994) 66:259–67. 10.1016/S0006-3495(94)80775-1 PMC1275686 8130344

[8] Alexander AL Lee JE Lazar M Field AS Diffusion tensor imaging of the brain. Neurotherapeutics (2007) 4:316–29. 10.1016/j.nurt.2007.05.011 PMC2041910 17599699

[9] Mighdoll MI Tao R Kleinman JE Hyde TM Myelin, myelin-related disorders, and psychosis. Schizophr Res (2015) 161:85–93. 10.1016/j.schres.2014.09.040 25449713

[10] Jones DK Knösche TR Turner R White matter integrity, fiber count, and other fallacies: the do's and don'ts of diffusion MRI. NeuroImage (2013) 73:239–54. 10.1016/j.neuroimage.2012.06.081 22846632

[11] Pasternak O Sochen N Gur Y Intrator N Assaf Y Free water elimination and mapping from diffusion MRI. Magn Reson Med (2009) 62:717–30. 10.1002/mrm.22055 19623619

[12] Oestreich LKL Lyall AE Pasternak O Kikinis Z Newell DT Savadjiev P Characterizing white matter changes in chronic schizophrenia: A free-water imaging multi-site study. Schizophr Res (2017) 189:153–61. 10.1016/j.schres.2017.02.006 PMC5552442 28190639

[13] Pasternak O Westin CF Bouix S Seidman LJ Goldstein JM Woo TU Excessive extracellular volume reveals a neurodegenerative pattern in schizophrenia onset. J Neurosci (2012) 32:17365–72. 10.1523/JNEUROSCI.2904-12.2012 PMC3549332 23197727

[14] Lyall AE Pasternak O Robinson DG Newell D Trampush JW Gallego JA Greater extracellular free-water in first-episode psychosis predicts better neurocognitive functioning. Mol Psychiatry (2018) 23:701–7. 10.1038/mp.2017.43 PMC5617750 28348381

[15] Pasternak O Westin CF Dahlben B Bouix S Kubicki M The extent of diffusion MRI markers of neuroinflammation and white matter deterioration in chronic schizophrenia. Schizophr Res (2015) 161:113–8. 10.1016/j.schres.2014.07.031 PMC4277709 25126717

[16] Rimol LM Hartberg CB Nesvåg R Fennema-Notestine C Hagler DJ Pung CJ Cortical thickness and subcortical volumes in schizophrenia and bipolar disorder. Biol Psychiatry (2010) 68:41–50. 10.1016/j.biopsych.2010.03.036 20609836

[17] Haukvik UK Westlye LT Morch-Johnsen L Jorgensen KN Lange EH Dale AM In vivo hippocampal subfield volumes in schizophrenia and bipolar disorder. Biol Psychiatry (2015) 77:581–8. 10.1016/j.biopsych.2014.06.020 25127742

[18] Nesvåg R Schaer M Haukvik UK Westlye LT Rimol LM Lange EH Reduced brain cortical folding in schizophrenia revealed in two independent samples. Schizophr Res (2014) 152:333–8. 10.1016/j.schres.2013.11.032 24365403

[19] Rimol LM Nesvåg R Hagler DJ Bergmann O Fennema-Notestine C Hartberg CB Cortical volume, surface area, and thickness in schizophrenia and bipolar disorder. Biol Psychiatry (2012) 71:552–60. 10.1016/j.biopsych.2011.11.026 22281121

[20] Ehrlich S Geisler D Yendiki A Panneck P Roessner V Calhoun VD Associations of white matter integrity and cortical thickness in patients with schizophrenia and healthy controls. Schizophr Bull (2014) 40:665–74. 10.1093/schbul/sbt056 PMC3984509 23661633

[21] Koch K Schultz CC Wagner G Schachtzabel C Reichenbach JR Sauer H Disrupted white matter connectivity is associated with reduced cortical thickness in the cingulate cortex in schizophrenia. Cortex J Devoted Study Nerv Syst Behav (2013) 49:722–9. 10.1016/j.cortex.2012.02.001 22402338

[22] Di Biase MA Cropley VL Cocchi L Fornito A Calamante F Ganella EP Linking cortical and connectional pathology in schizophrenia. Schizophr Bull (2018) 45: 911–23. 10.1093/schbul/sby121 PMC6581130 30215783

[23] Spoletini I Cherubini A Banfi G Rubino IA Peran P Caltagirone C Hippocampi, thalami, and accumbens microstructural damage in schizophrenia: a volumetry, diffusivity, and neuropsychological study. Schizophr Bull (2009) 37:118–30. 10.1093/schbul/sbp058 PMC3004185 19542526

[24] Ozcelik-Eroglu E Ertugrul A Oguz KK Has AC Karahan S Yazici MK Effect of clozapine on white matter integrity in patients with schizophrenia: a diffusion tensor imaging study. Psychiatry Res (2014) 223:226–35. 10.1016/j.pscychresns.2014.06.001 25012780

[25] Ebdrup BH Raghava JM Nielsen MØ Rostrup E Glenthøj B Frontal fasciculi and psychotic symptoms in antipsychotic-naive patients with schizophrenia before and after 6 weeks of selective dopamine D2/3 receptor blockade. J Psychiatry Neurosci JPN (2016) 41:133–41. 10.1503/jpn.150030 PMC4764482 26599135

[26] Ekholm B Ekholm A Adolfsson R Vares M Ösby U Sedvall GC Evaluation of diagnostic procedures in Swedish patients with schizophrenia and related psychoses. Nord J Psychiatry (2005) 59:457–64. 10.1080/08039480500360906 16316898

[27] Spitzer RL Williams JBW Gibbon M First MB Structured clinical interview for DSM-III-R - patient version (SCID-P). (New York: Biometrics Research Department, New York State Psychiatric Institute), (1988).

[28] American Psychiatric Association Diagnostic and Statistical Manual of Mental Disorders, International Version. 4th ed Washington DC: American Psychiatric Association (1995).

[29] Andreasen NC The scale for the assessment of negative symptoms (SANS). Iowa City, IA: University of Iowa (1983).

[30] Andreasen NC The scale for the assessment of positive symptoms (SAPS). Iowa City, IA: University of Iowa (1984).

[31] Pedersen G Hagtvet KA Karterud S Generalizability studies of the global assessment of functioning-split version. Compr Psychiatry (2007) 48:88–94. 10.1016/j.comppsych.2006.03.008 17145287

[32] Woods SW Chlorpromazine equivalent doses for the newer atypical antipsychotics. J Clin Psychiatry (2003) 64:663–7. 10.4088/JCP.v64n0607 12823080

[33] Andersson JLR Sotiropoulos SN An integrated approach to correction for off-resonance effects and subject movement in diffusion MR imaging. NeuroImage (2016) 125:1063–78. 10.1016/j.neuroimage.2015.10.019 PMC4692656 26481672

[34] Andersson JLR Graham MS Zsoldos E Sotiropoulos SN Incorporating outlier detection and replacement into a non-parametric framework for movement and distortion correction of diffusion MR images. NeuroImage (2016) 141:556–72. 10.1016/j.neuroimage.2016.06.058 27393418

[35] Tonnesen S Kaufmann T Doan NT Alnaes D Cordova-Palomera A Meer DV White matter aberrations and age-related trajectories in patients with schizophrenia and bipolar disorder revealed by diffusion tensor imaging. Sci Rep (2018) 8:14129. 10.1038/s41598-018-32355-9 30237410 PMC6147807

[36] Smith SM Jenkinson M Johansen-Berg H Rueckert D Nichols TE Mackay CE Tract-based spatial statistics: voxelwise analysis of multi-subject diffusion data. NeuroImage (2006) 31:1487–505. 10.1016/j.neuroimage.2006.02.024 16624579

[37] Jahanshad N Kochunov PV Sprooten E Mandl RC Nichols TE Almasy L Multi-site genetic analysis of diffusion images and voxelwise heritability analysis: a pilot project of the ENIGMA-DTI working group. NeuroImage (2013) 81:455–69. 10.1016/j.neuroimage.2013.04.061 PMC3729717 23629049

[38] Mori S Oishi K Jiang H Jiang L Li X Akhter K Stereotaxic white matter atlas based on diffusion tensor imaging in an ICBM template. NeuroImage (2008) 40:570–82. 10.1016/j.neuroimage.2007.12.035 PMC2478641 18255316

[39] Pasternak O Kelly S Sydnor VJ Shenton ME Advances in microstructural diffusion neuroimaging for psychiatric disorders. NeuroImage (2018) 182: 259–82. 10.1016/j.neuroimage.2018.04.051 PMC6420686 29729390

[40] Fischl B FreeSurfer. NeuroImage (2012) 62:774–81. 10.1016/j.neuroimage.2012.01.021 PMC3685476 22248573

[41] Zheng W Chee MW Zagorodnov V Improvement of brain segmentation accuracy by optimizing non-uniformity correction using N3. NeuroImage (2009) 48:73–83. 10.1016/j.neuroimage.2009.06.039 19559796

[42] Fischl B Salat DH Busa E Albert M Dieterich M Haselgrove C Whole brain segmentation: automated labeling of neuroanatomical structures in the human brain. Neuron (2002) 33:341–55. 10.1016/S0896-6273(02)00569-X 11832223

[43] Iglesias JE Augustinack JC Nguyen K Player CM Player A Wright M A computational atlas of the hippocampal formation using ex vivo, ultra-high resolution MRI: Application to adaptive segmentation of *in vivo* MRI. NeuroImage (2015) 115:117–37. 10.1016/j.neuroimage.2015.04.042 PMC4461537 25936807

[44] Saygin ZM Kliemann D Iglesias JE van der Kouwe AJW Boyd E Reuter M High-resolution magnetic resonance imaging reveals nuclei of the human amygdala: manual segmentation to automatic atlas. NeuroImage (2017) 155:370–82. 10.1016/j.neuroimage.2017.04.046 PMC5557007 28479476

[45] Tournier JD Mori S Leemans A Diffusion tensor imaging and beyond. Magn Reson Med (2011) 65:1532–56. 10.1002/mrm.22924 PMC3366862 21469191

[46] Royston P Remark AS R94: A Remark on Algorithm AS 181: The W-test for Normality. Appl Stat (1995) 44:547–51. 10.2307/2986146

[47] Nakagawa S Cuthill IC Effect size, confidence interval and statistical significance: a practical guide for biologists. Biol Rev Camb Philos Soc (2007) 82:591–605. 10.1111/j.1469-185X.2007.00027.x 17944619

[48] Benjamini Y Hochberg Y Controlling the False Discovery Rate - a Practical and Powerful Approach to Multiple Testing. J R Stat Soc Ser B-Methodol (1995) 57:289–300. 10.1111/j.2517-6161.1995.tb02031.x

[49] Catani M Dell'acqua F Thiebaut de Schotten M A revised limbic system model for memory, emotion and behaviour. Neurosci Biobehav Rev (2013) 37:1724–37. 10.1016/j.neubiorev.2013.07.001 23850593

[50] Barbas H General cortical and special prefrontal connections: principles from structure to function. Annu Rev Neurosci (2015) 38:269–89. 10.1146/annurev-neuro-071714-033936 25897871

[51] Pettersson-Yeo W Allen P Benetti S McGuire P Mechelli A Dysconnectivity in schizophrenia: Where are we now? Neurosci Biobehav Rev (2011) 35:1110–24. 10.1016/j.neubiorev.2010.11.004 21115039

[52] van den Heuvel MP Fornito A Brain networks in schizophrenia. Neuropsychol Rev (2014) 24:32–48. 10.1007/s11065-014-9248-7 24500505

[53] Schmitt A Hasan A Gruber O Falkai P Schizophrenia as a disorder of disconnectivity. Eur Arch Psychiatry Clin Neurosci (2011) 261:150. 10.1007/s00406-011-0242-2 PMC3207137 21866371

[54] Jørgensen KN Nesvåg R Gunleiksrud S Raballo A Jönsson EG Agartz I First- and second-generation antipsychotic drug treatment and subcortical brain morphology in schizophrenia. Eur Arch Psychiatry Clin Neurosci (2016) 266:451–60. 10.1007/s00406-015-0650-9 26547434

[55] Westlye LT Alnæs D van der Meer D Kaufmann T Andreassen OA Population-Based Mapping of Polygenic Risk for Schizophrenia on the Human Brain: New Opportunities to Capture the Dimensional Aspects of Severe Mental Disorders. Biol Psychiatry (2019) 86:499–501. 10.1016/j.biopsych.2019.08.001 31521208

[56] Gurholt TP Haukvik UK Lonning V Jönsson EG Pasternak O Agartz I Microstructural white matter and links with subcortical structures in chronic schizophrenia: A free-water imaging approach. bioRxiv [Preprint] (2019) 621482. 10.1101/621482 PMC7057718 32180735

