# Supplementary material for: Microstructural White Matter and Links With Subcortical Structures in Chronic Schizophrenia: A Free-Water Imaging Approach
Source: Front Psychiatry. 2020 Feb 27;11:56. doi: 10.3389/fpsyt.2020.00056 (PMC7057718; doi:10.3389/fpsyt.2020.00056)
Supplement: Supplementary file 1 [file DataSheet_1.pdf]

Supplemental Material

Table of Contents

Table S1: The Cohen's d effect size of patient-control differences for FA_t, AD_t, RD_t and FW.

Table S2: The Cohen's d effect size of patient-control differences for FA, MD, RD and AD (standard DTI method).

Table S3: The Cohen's d effect size of CPZ on all ROIs in patients.

Table S4: The Cohen's d effect size of total SAPS on all ROIs in patients.

Table S5*:* The Cohen's d effect size of total SANS on all ROIs in patients.

Table S6: Interaction effects between patient status and subcortical structures on the left ACR.

Table S7: Interaction effects between patient status and subcortical structures on the right ACR.

Table S8: Interaction effects between patient status and subcortical structures on the left ALIC.

Table S9: Interaction effects between patient status and subcortical structures on the fornix.

Table S1: The Cohen's d effect size of patient-control differences for FA_t, AD_t, RD_t and FW.

	FA_t			AD_t			RD_t			FW		
**ROI**	**d**	**t**	**p**	**d**	**t**	**p**	**d**	**t**	**p**	**d**	**t**	**p**
**R ACR**	−0.96	−3.88	**0.0002**	−0.94	−3.78	**0.0003**	0.94	3.79	**0.0003**	0.41	1.65	0.1038
**L ACR**	−0.74	−2.98	**0.0040**	−0.75	−3.02	**0.0035**	0.71	2.87	**0.0055**	0.34	1.37	0.1738
**L ALIC**	−0.69	−2.79	**0.0069**	−0.71	−2.85	**0.0058**	0.70	2.81	**0.0064**	−0.34	−1.37	0.1748
**Average**	−0.61	−2.48	0.0156	−0.64	−2.57	0.0124	0.59	2.36	0.0211	0.34	1.39	0.1685
**GCC**	−0.60	−2.40	0.0189	−0.29	−1.16	0.2521	0.49	1.98	0.0514	0.18	0.71	0.4784
**L SS**	−0.56	−2.24	0.0281	−0.54	−2.17	0.0332	0.54	2.16	0.0343	0.25	1.03	0.3089
**CC**	−0.50	−2.00	0.0496	−0.25	−1.01	0.3147	0.37	1.49	0.1401	0.24	0.97	0.3342
**BCC**	−0.42	−1.70	0.0932	−0.26	−1.04	0.3020	0.38	1.53	0.1300	0.24	0.99	0.3268
**L PTR**	−0.41	−1.66	0.1009	−0.37	−1.49	0.1414	0.40	1.61	0.1118	0.25	1.02	0.3136
**L IC**	−0.37	−1.50	0.1392	−0.20	−0.82	0.4149	0.28	1.13	0.2622	0.05	0.22	0.8281
**R ALIC**	−0.36	−1.47	0.1458	−0.37	−1.49	0.1414	0.35	1.42	0.1610	0.02	0.07	0.9404
**R CR**	−0.36	−1.47	0.1469	−0.31	−1.24	0.2176	0.31	1.23	0.2222	0.34	1.36	0.1777
**L SLF**	−0.34	−1.35	0.1806	−0.32	−1.29	0.2001	0.27	1.11	0.2721	0.45	1.84	0.0709
**R SS**	−0.29	−1.19	0.2400	−0.24	−0.97	0.3333	0.26	1.03	0.3045	0.12	0.59	0.6164
**FX**	−0.26	−1.06	0.2933	0.19	0.77	0.4449	0.82	3.32	**0.0015**	0.25	1.03	0.3083
**R SLF**	−0.25	−1.02	0.3118	−0.25	−1.00	0.3220	0.22	0.88	0.3803	0.26	1.04	0.3022
**L RLIC**	−0.21	−0.84	0.4041	−0.11	−0.43	0.6693	0.12	0.47	0.6399	0.37	1.48	0.1431
**L CR**	−0.19	−0.75	0.4563	−0.18	−0.71	0.4813	0.15	0.61	0.5415	0.36	1.46	0.1494
**L CGH**	−0.18	−0.71	0.4778	−0.18	−0.71	0.4785	0.28	1.13	0.2625	−0.12	−0.49	0.6271
**R CGC**	−0.16	−0.66	0.5124	−0.21	−0.84	0.4015	0.15	0.62	0.5360	0.24	0.97	0.3362
**R RLIC**	−0.15	−0.59	0.5593	−0.09	−0.35	0.7290	0.1	0.42	0.6785	0.16	0.65	0.5172
**L CST**	−0.14	−0.58	0.5613	−0.51	−2.04	0.0451	−0.22	−0.9	0.3734	−0.22	−0.91	0.3680
**L SFO**	−0.13	−0.54	0.5898	−0.12	−0.5	0.6208	0.10	0.41	0.6828	−0.14	−0.55	0.5842
**R FXST**	−0.12	−0.46	0.6435	−0.01	−0.03	0.9793	0.10	0.42	0.6755	−0.25	−1.02	0.3136
**R PTR**	−0.11	−0.43	0.6659	−0.06	−0.23	0.8211	0.07	0.3	0.7654	0.24	0.97	0.3380
**L CGC**	−0.10	−0.39	0.6983	−0.11	−0.43	0.6686	0.12	0.48	0.6297	0.44	1.76	0.0825
**R EC**	−0.10	−0.41	0.6853	−0.21	−0.86	0.3934	0.20	0.81	0.4232	0.08	0.32	0.7533
**L FXST**	−0.10	−0.39	0.6982	−0.03	−0.13	0.8999	0.08	0.34	0.7334	0.54	2.20	0.0313
**R IC**	−0.10	−0.41	0.6859	0.02	0.08	0.9335	0.03	0.14	0.8886	0.07	0.27	0.7884
**SCC**	−0.06	−0.23	0.8198	−0.09	−0.38	0.7050	−0.09	−0.37	0.7135	0.19	0.76	0.4513
**R CGH**	−0.03	−0.11	0.9092	−0.07	−0.27	0.7870	0.03	0.12	0.9022	0.02	0.06	0.9497
**L EC**	−0.03	−0.11	0.9138	−0.10	−0.41	0.6821	0.08	0.33	0.7423	0.01	0.04	0.9681
**R PCR**	0	0	0.9977	0.04	0.16	0.8709	−0.08	−0.34	0.7342	0.24	0.98	0.3290
**L PLIC**	0.01	0.06	0.9528	0.16	0.64	0.5237	−0.04	−0.17	0.8692	0.11	0.44	0.6618
**R UNC**	0.04	0.15	0.8818	0.08	0.31	0.7550	0	−0.01	0.9942	0.15	0.62	0.5369
**L PCR**	0.05	0.22	0.8260	0.06	0.26	0.7965	−0.07	−0.3	0.7672	0.34	1.38	0.1720
**L IFO**	0.09	0.38	0.7086	0.06	0.25	0.7999	−0.19	−0.76	0.4518	−0.42	−1.69	0.0948
**R CST**	0.12	0.49	0.6243	0.01	0.04	0.9688	−0.38	−1.54	0.1283	0.02	0.09	0.9257
**L UNC**	0.12	0.49	0.6261	0.03	0.12	0.9071	−0.06	−0.23	0.8218	−0.07	−0.29	0.7705
**R SCR**	0.14	0.55	0.5849	0.13	0.54	0.5928	−0.13	−0.53	0.5952	0.23	0.93	0.3546
**R SFO**	0.18	0.74	0.4589	0.13	0.52	0.6070	−0.13	−0.52	0.6031	−0.05	−0.19	0.8524
**R IFO**	0.20	0.82	0.4156	0.22	0.9	0.3693	−0.21	−0.83	0.4098	−0.04	−0.18	0.8601
**R PLIC**	0.26	1.06	0.2939	0.37	1.5	0.1375	−0.29	−1.15	0.2526	0	−0.01	0.9941
**L SCR**	0.27	1.1	0.2757	0.23	0.94	0.3519	−0.25	−1.01	0.3149	0.32	1.30	0.1966

Notes: Results from regression *Model 1*: the effect of patient status on ROIs after adjusting for age, sex and average motion. The ROIs are ordered by FA_t effect size. ROIs that pass FDR threshold, $p \leq 0.0109$, are indicated in bold. *Abbreviations:* ACR: anterior corona radiata, AD_t: FW adjusted axial diffusivity, ALIC: anterior limb of internal capsule, Average: average of FA_t, AD_t, RD_t, and FW, respectively. BCC: body of corpus callosum, CC: corpus callosum, CGC: cingulum, CGH: cingulum hippocampal portion, CR: corona radiata, CST: corticospinal tract, EC: external capsule, FA_t: FW adjusted fractional anisotropy, FW: free-water, FX: fornix, FXST: fornix stria terminalis, GCC: genu of corpus callosum, IC: internal capsule, IFO: inferior fronto occipital fasciculus, L: Left, PCR: posterior corona radiata, PLIC: posterior limb of internal capsule, PTR: posterior thalamic radiation, R: Right, RD_t: FW adjusted Radial diffusivity, RLIC: retrolenticular part of IC, ROI: region of interest, SCC: splenium of corpus callosum, SCR: superior corona radiata, SFO: superior fronto-occipital fasciculus, SLF: superior longitudinal fasciculus, SS: sagittal stratum, UNC: uncinate.

Table S2: The Cohen's d effect size of patient-control differences for FA, MD, RD and AD (standard DTI method).

	FA			AD			RD			MD		
**ROI**	**d**	**t**	**p**	**d**	**t**	**p**	**d**	**t**	**p**	**d**	**t**	**p**
**R ACR**	−0.88	−3.55	**0.0007**	−0.15	−0.62	0.5361	0.66	2.65	**0.0100**	0.42	1.69	0.0962
**L ACR**	−0.62	−2.48	0.0155	−0.09	−0.35	0.7304	0.52	2.09	0.0400	0.36	1.46	0.1493
**L SLF**	−0.60	−2.44	0.0174	0.08	0.34	0.7382	0.52	2.11	0.0388	0.43	1.72	0.0894
**Average**	−0.56	−2.27	0.0266	0.08	0.34	0.7372	0.48	1.93	0.0584	0.38	1.52	0.1327
**R CR**	−0.49	−1.97	0.0534	0.09	0.36	0.7196	0.44	1.77	0.0811	0.34	1.37	0.1745
**L SS**	−0.48	−1.93	0.0583	−0.21	−0.85	0.3966	0.43	1.73	0.0889	0.26	1.04	0.3034
**FX**	−0.43	−1.72	0.0895	0.24	0.96	0.3395	0.24	0.95	0.3453	0.24	0.96	0.3411
**R ALIC**	−0.39	−1.57	0.1215	−0.16	−0.66	0.5126	0.13	0.52	0.6080	0.01	0.04	0.9680
**L ALIC**	−0.37	−1.50	0.1382	−0.64	−2.57	0.0125	0.01	0.02	0.9834	−0.34	−1.36	0.1784
**L PTR**	−0.36	−1.44	0.1559	−0.07	−0.29	0.7709	0.33	1.34	0.1854	0.24	0.95	0.3455
**L CR**	−0.34	−1.35	0.1804	0.20	0.82	0.4146	0.39	1.56	0.1238	0.37	1.48	0.1433
**BCC**	−0.32	−1.29	0.2020	0.06	0.23	0.8205	0.33	1.33	0.1892	0.28	1.14	0.2583
**L RLIC**	−0.32	−1.30	0.1968	0.18	0.73	0.4656	0.36	1.46	0.1478	0.38	1.54	0.1278
**R SLF**	−0.32	−1.30	0.1966	0.03	0.11	0.9152	0.33	1.34	0.1834	0.25	0.99	0.3250
**CC**	−0.30	−1.19	0.2380	0.03	0.12	0.9060	0.36	1.46	0.1483	0.28	1.13	0.2640
**R SS**	−0.30	−1.20	0.2360	−0.09	−0.37	0.7099	0.24	0.97	0.3363	0.14	0.57	0.5730
**GCC**	−0.26	−1.06	0.2909	−0.02	−0.09	0.9298	0.36	1.46	0.1482	0.25	1.01	0.3171
**L IC**	−0.26	−1.04	0.3023	−0.06	−0.25	0.8027	0.18	0.74	0.4633	0.10	0.42	0.6756
**L FXST**	−0.23	−0.93	0.3541	0.24	0.99	0.3267	0.44	1.79	0.0775	0.62	2.50	0.0147
**L PCR**	−0.22	−0.89	0.3781	0.27	1.09	0.2787	0.27	1.09	0.2776	0.33	1.33	0.1869
**R PCR**	−0.22	−0.87	0.3878	0.18	0.75	0.4580	0.20	0.81	0.4225	0.23	0.92	0.3633
**R RLIC**	−0.20	−0.80	0.4279	0.08	0.31	0.7571	0.20	0.81	0.4183	0.21	0.85	0.4002
**R CGC**	−0.17	−0.67	0.5032	−0.02	−0.08	0.9385	0.29	1.16	0.2503	0.24	0.96	0.3402
**R EC**	−0.15	−0.61	0.5436	−0.04	−0.17	0.8631	0.13	0.51	0.6135	0.07	0.30	0.7631
**L CGC**	−0.11	−0.43	0.6696	0.11	0.46	0.6458	0.38	1.52	0.1323	0.42	1.70	0.0939
**R IC**	−0.11	−0.45	0.6516	0.12	0.50	0.6193	0.07	0.29	0.7720	0.12	0.5	0.6173
**L SFO**	−0.11	−0.43	0.6703	−0.11	−0.43	0.6652	−0.09	−0.36	0.7175	−0.12	−0.49	0.6248
**L CGH**	−0.10	−0.42	0.6794	−0.25	−1.00	0.3225	−0.03	−0.14	0.8915	−0.16	−0.64	0.5220
**R PTR**	−0.10	−0.41	0.6811	0.14	0.58	0.5610	0.25	1.01	0.3181	0.24	0.96	0.3381
**R UNC**	−0.06	−0.23	0.8202	0.15	0.62	0.5385	0.13	0.53	0.6010	0.17	0.67	0.5041
**SCC**	−0.05	−0.21	0.8315	0.02	0.10	0.9205	0.19	0.77	0.4434	0.15	0.6	0.5525
**L EC**	−0.03	−0.11	0.9142	−0.03	−0.11	0.9108	0.03	0.14	0.8914	0.01	0.06	0.9522
**R CGH**	0.03	0.11	0.9118	0.08	0.31	0.7583	0.07	0.28	0.7795	0.11	0.45	0.6537
**L CST**	0.03	0.13	0.8950	−0.40	−1.63	0.1073	−0.16	−0.65	0.5210	−0.43	−1.72	0.0898
**L PLIC**	0.04	0.14	0.8860	0.25	0.99	0.3251	0.05	0.21	0.8327	0.19	0.78	0.4402
**R SCR**	0.05	0.19	0.8518	0.22	0.89	0.3771	0.14	0.56	0.5775	0.23	0.94	0.3496
**R FXST**	0.06	0.26	0.7958	−0.01	−0.03	0.9758	−0.10	−0.39	0.7004	−0.09	−0.38	0.7065
**L UNC**	0.07	0.28	0.7803	−0.04	−0.16	0.8714	−0.12	−0.48	0.6294	−0.11	−0.45	0.6522
**L SCR**	0.09	0.37	0.7103	0.35	1.39	0.1679	0.13	0.54	0.5932	0.31	1.27	0.2091
**R SFO**	0.19	0.76	0.4483	0.11	0.46	0.6490	−0.14	−0.55	0.5866	−0.03	−0.13	0.8983
**R IFO**	0.25	1.00	0.3195	0.17	0.69	0.4926	−0.15	−0.59	0.5544	0	−0.02	0.9872
**R PLIC**	0.27	1.11	0.2730	0.41	1.64	0.1058	−0.18	−0.71	0.4805	0.14	0.58	0.5658
**R CST**	0.29	1.16	0.2500	0.14	0.56	0.5803	−0.16	−0.63	0.5313	−0.01	−0.02	0.9831
**L IFO**	0.34	1.37	0.1744	−0.09	−0.35	0.7273	−0.38	−1.53	0.1299	−0.44	−1.78	0.0799

Notes: Results from regression *Model 1*: the effect of patient status on ROIs after adjusting for age, sex and average motion. The ROIs are ordered by FA effect sizes. ROIs that pass FDR threshold, $p \leq 0.0109$, are indicated in bold. *Abbreviations:* ACR: anterior corona radiata, AD: axial diffusivity, ALIC: anterior limb of internal capsule, BCC: body of corpus callosum, CC: corpus callosum, CGC: cingulum, CGH: cingulum hippocampal portion, CR: corona radiata, CST: corticospinal tract, EC: external capsule, FA: fractional anisotropy, FX: fornix, FXST: fornix stria terminalis, GCC: genu of corpus callosum, IC: internal capsule, IFO: inferior fronto occipital fasciculus, MD: mean diffusivity, PCR: posterior corona radiata, PLIC: posterior limb of internal capsule, PTR: posterior thalamic radiation, RD: radial diffusivity, RLIC: retrolenticular part of IC, ROI: region of interest, SCC: splenium of corpus callosum, SCR: superior corona radiata, SFO: superior fronto-occipital fasciculus, SLF: superior longitudinal fasciculus, SS: sagittal stratum, UNC: uncinate.

Table S3: The Cohen's d effect size of CPZ on all ROIs in patients.

ROI	FA_t d	t	p	AD_t d	t	p	RD_t d	t	p	FW d	t	p
R ALIC	−0.84	−2.10	0.0455	−0.87	−2.18	0.0392	0.70	1.75	0.0918	−0.19	−0.47	0.6450
L SLF	−0.54	−1.35	0.1882	−0.38	−0.94	0.3545	0.42	1.05	0.3016	−0.15	−0.36	0.7195
R CR	−0.52	−1.31	0.2026	−0.43	−1.07	0.2948	0.47	1.18	0.2484	−0.31	−0.77	0.4513
R UNC	−0.52	−1.30	0.2054	−0.31	−0.79	0.4397	0.42	1.06	0.3001	−0.20	−0.50	0.6180
L CR	−0.49	−1.23	0.2306	−0.50	−1.25	0.2213	0.49	1.21	0.2365	−0.22	−0.54	0.5935
Average	−0.48	−1.20	0.2421	−0.60	−1.49	0.1481	0.32	0.80	0.4289	−0.25	−0.62	0.5394
L SCR	−0.48	−1.19	0.2441	−0.44	−1.10	0.2805	0.44	1.10	0.2830	−0.38	−0.95	0.3530
R SCR	−0.43	−1.07	0.2958	−0.36	−0.91	0.3713	0.37	0.92	0.3649	−0.30	−0.76	0.4541
L PTR	−0.42	−1.05	0.3041	−0.27	−0.68	0.5050	0.34	0.86	0.4003	−0.41	−1.03	0.3147
R ACR	−0.35	−0.89	0.3840	−0.36	−0.91	0.3728	0.36	0.89	0.3821	−0.16	−0.40	0.6946
R IC	−0.35	−0.88	0.3885	−0.28	−0.70	0.4919	0.25	0.62	0.5408	−0.27	−0.68	0.5021
R PCR	−0.35	−0.88	0.3852	−0.21	−0.53	0.5974	0.33	0.82	0.4189	−0.52	−1.30	0.2056
SCC	−0.35	−0.86	0.3961	−0.25	−0.63	0.5376	0.24	0.60	0.5517	−0.43	−1.07	0.2938
CC	−0.32	−0.81	0.4261	−0.35	−0.87	0.3939	0.26	0.65	0.5234	−0.34	−0.84	0.4098
L IFO	−0.30	−0.74	0.4664	−0.13	−0.33	0.7460	0.24	0.59	0.5580	0.09	0.23	0.8209
L SS	−0.28	−0.69	0.4959	−0.32	−0.80	0.4295	0.27	0.68	0.5017	−0.42	−1.05	0.3032
L ACR	−0.26	−0.65	0.5231	−0.33	−0.82	0.4192	0.31	0.79	0.4393	0	0	0.9989
R PLIC	−0.25	−0.64	0.5311	−0.20	−0.49	0.6279	0.21	0.52	0.6077	0.02	0.04	0.9688
L CST	−0.24	−0.60	0.5529	−0.29	−0.74	0.4677	0.35	0.88	0.3849	−0.11	−0.28	0.7817
L PCR	−0.24	−0.60	0.5527	−0.24	−0.59	0.5598	0.22	0.55	0.5896	−0.44	−1.10	0.2834
R SFO	−0.24	−0.61	0.5506	−0.31	−0.77	0.4477	0.29	0.72	0.4802	−0.46	−1.16	0.2564
GCC	−0.23	−0.59	0.5628	−0.14	−0.35	0.7303	0.23	0.56	0.5780	−0.01	−0.02	0.9859
L ALIC	−0.22	−0.54	0.5908	−0.32	−0.79	0.4354	0.20	0.49	0.6275	−0.04	−0.10	0.9248
BCC	−0.21	−0.51	0.6116	−0.36	−0.89	0.3831	0.15	0.38	0.7081	−0.40	−1.00	0.3275
R IFO	−0.20	−0.51	0.6150	−0.18	−0.45	0.6575	0.16	0.40	0.6940	−0.32	−0.80	0.4335
L PLIC	−0.19	−0.49	0.6302	−0.29	−0.73	0.4704	0.12	0.30	0.7692	−0.78	−1.94	0.0640
R SLF	−0.16	−0.39	0.7016	0.03	0.09	0.9326	0	0	0.9989	−0.05	−0.13	0.8973
L UNC	−0.15	−0.37	0.7120	−0.08	−0.20	0.8437	0.05	0.12	0.9061	−0.10	−0.25	0.8047
L CGC	−0.14	−0.34	0.7374	−0.02	−0.05	0.9624	0.19	0.48	0.6383	−0.42	−1.04	0.3073
L IC	−0.14	−0.36	0.7234	−0.19	−0.46	0.6464	0.07	0.17	0.8668	−0.48	−1.21	0.2379
L FXST	−0.09	−0.22	0.8276	−0.08	−0.19	0.8525	0.05	0.14	0.8926	−0.40	−1.01	0.3215
L EC	−0.05	−0.13	0.8960	−0.10	−0.24	0.8140	0.09	0.22	0.8256	0.10	0.24	0.8088
R EC	0	0	0.9987	−0.04	−0.11	0.9139	0.01	0.02	0.9824	−0.48	−1.20	0.2431
R SS	0.01	0.03	0.9799	−0.07	−0.16	0.8719	−0.01	−0.02	0.9847	−0.44	−1.10	0.2834
R CGC	0.04	0.10	0.9215	0.12	0.30	0.7702	0.02	0.06	0.9560	−0.24	−0.61	0.5504
L RLIC	0.08	0.21	0.8363	0.17	0.43	0.6718	−0.15	−0.37	0.7133	−0.32	−0.81	0.4254
R PTR	0.09	0.22	0.8297	0.05	0.12	0.9056	−0.09	−0.23	0.8208	−0.33	−0.83	0.4168
R CST	0.14	0.34	0.7358	0.02	0.05	0.9621	−0.02	−0.05	0.9589	−0.39	−0.97	0.3419
L SFO	0.14	0.36	0.7240	0.08	0.19	0.8491	−0.21	−0.52	0.6080	−0.43	−1.08	0.2920
R RLIC	0.19	0.48	0.6333	0.21	0.53	0.5974	−0.21	−0.53	0.6039	−0.47	−1.18	0.2484
FX	0.20	0.51	0.6162	0.25	0.63	0.5320	−0.12	−0.29	0.7718	−0.10	−0.24	0.8096
L CGH	0.22	0.56	0.5825	0.16	0.39	0.6972	−0.28	−0.69	0.4973	−0.45	−1.12	0.2727
R FXST	0.29	0.72	0.4777	0.26	0.64	0.5294	−0.24	−0.61	0.5464	−0.43	−1.07	0.2934
R CGH	0.43	1.08	0.2912	0.34	0.84	0.4094	−0.46	−1.15	0.2622	−0.31	−0.77	0.4486

Notes: Results from regression model that investigates the effect of CPZ on ROIs after adjusting for age, sex and average motion. The ROIs are ordered by FA_t effect size. ROIs that passed the FDR threshold, $p \leq 0.0109$, are indicated in bold. *Abbreviations:* ACR: anterior corona radiata, AD: axial diffusivity, ALIC: anterior limb of internal capsule, BCC: body of corpus callosum, CC: corpus callosum, CGC: cingulum, CGH: cingulum hippocampal portion, CR: corona radiata, CST: corticospinal tract, EC: external capsule, FA: fractional anisotropy, FX: fornix, FXST: fornix stria terminalis, GCC: genu of corpus callosum, IC: internal capsule, IFO: inferior fronto occipital fasciculus, MD: mean diffusivity, PCR: posterior corona radiata, PLIC: posterior limb of internal capsule, PTR: posterior thalamic radiation, RD: radial diffusivity, RLIC: retrolenticular part of IC, ROI: region of interest, SCC: splenium of corpus callosum, SCR: superior corona radiata, SFO: superior fronto-occipital fasciculus, SLF: superior longitudinal fasciculus, SS: sagittal stratum, UNC: uncinate.

Table S4: The Cohen's d effect size of total SAPS on all ROIs in patients.

ROI	FA_t			AD_t			RD_t			FW		
	d	t	p	d	t	p	d	t	p	d	t	p
L CGH	−0.83	−2.06	0.0497	−0.77	−1.91	0.0672	0.69	1.72	0.0981	0.73	1.84	0.0784
R RLIC	−0.83	−2.08	0.0478	−0.73	−1.83	0.0794	0.78	1.95	0.0623	0.72	1.79	0.0859
L PTR	−0.79	−1.99	0.0580	−0.77	−1.93	0.0656	0.79	1.97	0.0601	0.80	2.00	0.0566
L IC	−0.65	−1.63	0.1156	−0.56	−1.39	0.1769	0.65	1.63	0.1156	0.42	1.05	0.3018
R IC	−0.63	−1.59	0.1253	−0.45	−1.13	0.2710	0.69	1.74	0.0947	0.48	1.19	0.2451
R SS	−0.62	−1.55	0.1326	−0.51	−1.28	0.2116	0.63	1.56	0.1303	1.09	2.73	0.0113
GCC	−0.58	−1.46	0.1571	−0.19	−0.47	0.6395	0.59	1.47	0.1552	0.09	0.24	0.8143
R PTR	−0.58	−1.44	0.1616	−0.50	−1.25	0.2244	0.64	1.60	0.1229	1.20	2.99	**0.0061**
L ALIC	−0.57	−1.42	0.1691	−0.73	−1.81	0.0817	0.64	1.60	0.1222	−0.15	−0.37	0.7140
R SCR	−0.55	−1.36	0.1849	−0.51	−1.26	0.2180	0.50	1.26	0.2205	0.64	1.61	0.1209
R CGH	−0.53	−1.32	0.1991	−0.70	−1.74	0.0942	0.64	1.60	0.1217	0.08	0.20	0.8457
R PCR	−0.53	−1.32	0.2002	−0.35	−0.88	0.3858	0.52	1.30	0.2049	0.84	2.09	0.0467
L ACR	−0.51	−1.27	0.2148	−0.59	−1.48	0.1525	0.55	1.38	0.1783	0.63	1.57	0.1289
L RLIC	−0.49	−1.23	0.2294	−0.47	−1.17	0.2544	0.48	1.20	0.2411	0.66	1.65	0.1121
L CR	−0.48	−1.21	0.2375	−0.45	−1.14	0.2669	0.44	1.10	0.2818	0.82	2.05	0.0511
L PLIC	−0.48	−1.19	0.2452	−0.26	−0.66	0.5168	0.49	1.24	0.2281	0.27	0.67	0.5102
R CR	−0.40	−1.01	0.3211	−0.37	−0.92	0.3646	0.41	1.02	0.3155	0.65	1.62	0.1186
CC	−0.39	−0.96	0.3445	0	0	0.9963	0.37	0.93	0.3603	0.34	0.84	0.4068
L PCR	−0.37	−0.92	0.3675	−0.27	−0.68	0.5021	0.29	0.74	0.4689	0.78	1.94	0.0634
Average	−0.34	−0.86	0.3969	−0.21	−0.54	0.5967	0.53	1.33	0.1943	0.58	1.44	0.1614
R PLIC	−0.31	−0.78	0.4420	−0.14	−0.35	0.7326	0.35	0.88	0.3880	0.17	0.43	0.6684
R SFO	−0.23	−0.59	0.5625	−0.24	−0.61	0.5474	0.25	0.62	0.5407	0.67	1.68	0.1045
L SCR	−0.21	−0.52	0.6056	−0.18	−0.45	0.6597	0.17	0.44	0.6655	1.00	2.50	0.0194
BCC	−0.20	−0.51	0.6169	0.08	0.19	0.8525	0.17	0.41	0.6821	0.43	1.06	0.2973
SCC	−0.19	−0.48	0.6322	0.08	0.20	0.8407	0.24	0.60	0.5536	0.26	0.65	0.5210
R ALIC	−0.18	−0.45	0.6574	−0.18	−0.45	0.6594	0.42	1.05	0.3047	0.29	0.73	0.4724
R CGC	−0.18	−0.44	0.6613	−0.30	−0.75	0.4596	0.21	0.52	0.6081	0.46	1.14	0.2658
L SFO	−0.16	−0.39	0.6977	−0.04	−0.11	0.9159	0.25	0.63	0.5363	0.19	0.47	0.6427
L EC	−0.13	−0.32	0.7520	−0.11	−0.27	0.7892	0.12	0.29	0.7721	0.40	1.01	0.3221
L UNC	−0.08	−0.19	0.8517	−0.06	−0.16	0.8733	0.08	0.21	0.8365	0.49	1.22	0.2354
L SS	−0.07	−0.18	0.8592	−0.04	−0.10	0.9177	0.07	0.18	0.8608	1.16	2.89	**0.0078**
R EC	−0.06	−0.14	0.8915	−0.04	−0.10	0.9201	0.02	0.06	0.9508	0.11	0.28	0.7814
FX	−0.05	−0.13	0.8956	0.08	0.2	0.8417	0.82	2.06	0.0499	−0.37	−0.91	0.3698
L FXST	−0.04	−0.11	0.9151	−0.14	−0.36	0.7224	0.07	0.17	0.8665	0.36	0.90	0.3748
R UNC	−0.04	−0.10	0.9187	0.02	0.04	0.9697	0.04	0.10	0.9195	0.79	1.97	0.0597
R FXST	0	0.01	0.9957	0.05	0.13	0.8999	0.17	0.43	0.6710	0.33	0.83	0.4164
L CGC	0.03	0.08	0.9385	0.07	0.17	0.8629	−0.05	−0.13	0.9013	0.55	1.37	0.1838
L IFO	0.09	0.23	0.8166	0.08	0.21	0.8353	−0.26	−0.65	0.5205	−0.23	−0.57	0.5710
R ACR	0.11	0.29	0.7778	0.06	0.15	0.8837	−0.07	−0.18	0.8547	0.47	1.18	0.2507
R SLF	0.25	0.62	0.5403	0.36	0.89	0.3820	−0.28	−0.70	0.4922	0.75	1.88	0.0713
L SLF	0.43	1.07	0.2934	0.60	1.50	0.1463	−0.50	−1.26	0.2198	0.90	2.24	0.0343
R IFO	0.55	1.38	0.1805	0.61	1.53	0.1387	−0.64	−1.61	0.1200	0.31	0.78	0.4422
L CST	0.83	2.07	0.0489	0.76	1.89	0.0706	−0.85	−2.12	0.044	0.01	0.03	0.9749
R CST	1.05	2.62	0.0149	0.74	1.84	0.0779	−0.69	−1.72	0.0982	−0.19	−0.48	0.6341

Notes: Results from regression model that investigates the effect of total SAPS on ROIs after adjusting for age, sex and average motion. The ROIs are ordered by ascending effect sizes for FA_t. ROIs that pass FDR threshold, $p \leq 0.0109$, are indicated in bold.
Abbreviations: ACR: anterior corona radiata, AD: axial diffusivity, ALIC: anterior limb of internal capsule, BCC: body of corpus callosum, CC: corpus callosum, CGC: cingulum, CGH: cingulum hippocampal portion, CR: corona radiata, CST: corticospinal tract, EC: external capsule, FA: fractional anisotropy, FX: fornix, FXST: fornix stria terminalis, GCC: genu of corpus callosum, IC: internal capsule, IFO: inferior fronto occipital fasciculus, MD: mean diffusivity, PCR: posterior corona radiata, PLIC: posterior limb of internal capsule, PTR: posterior thalamic radiation, RD: radial diffusivity, RLIC: retrolenticular part of IC, ROI: region of interest, SCC: splenium of corpus callosum, SCR: superior corona radiata, SFO: superior fronto-occipital fasciculus, SLF: superior longitudinal fasciculus, SS: sagittal stratum, UNC: uncinate.

Table S5: The Cohen's d effect size of total SANS on all ROIs in patients.

ROI	FA_t			AD_t			RD_t			FW		
	d	t	p	d	t	p	d	t	p	d	t	p
**R ALIC**	−1.14	−2.84	**0.0088**	−0.97	−2.42	0.0233	1.20	2.99	**0.0061**	0.29	0.72	0.4757
**R IC**	−0.99	−2.48	0.0203	−0.65	−1.64	0.1142	0.96	2.39	0.0245	0.57	1.43	0.1641
**L ALIC**	−0.94	−2.35	0.0271	−0.82	−2.06	0.0498	0.89	2.22	0.0360	−0.07	−0.18	0.8611
**GCC**	−0.86	−2.15	0.0411	−0.27	−0.68	0.5025	0.97	2.41	0.0234	0.10	0.25	0.8070
**R CGC**	−0.68	−1.71	0.0996	−0.60	−1.51	0.1448	0.69	1.71	0.0990	0.11	0.28	0.7780
**R CGH**	−0.65	−1.62	0.1189	−0.66	−1.65	0.1117	0.64	1.60	0.1215	−0.44	−1.10	0.2810
**L IC**	−0.65	−1.63	0.1146	−0.45	−1.14	0.2670	0.64	1.61	0.1202	0.55	1.38	0.1812
**L SCR**	−0.65	−1.62	0.1183	−0.61	−1.52	0.1414	0.61	1.51	0.1424	0.27	0.67	0.5070
**L CR**	−0.54	−1.34	0.1926	−0.54	−1.35	0.1903	0.52	1.30	0.2067	0.33	0.82	0.4179
**R RLIC**	−0.53	−1.34	0.1935	−0.48	−1.19	0.2439	0.52	1.30	0.2062	0.74	1.86	0.0749
**L CGH**	−0.51	−1.27	0.2154	−0.48	−1.19	0.2445	0.40	1.00	0.3251	−0.24	−0.60	0.5512
**R ACR**	−0.50	−1.25	0.2231	−0.55	−1.37	0.1817	0.55	1.38	0.1811	0.29	0.72	0.4810
**R CR**	−0.48	−1.21	0.2386	−0.49	−1.21	0.2362	0.54	1.36	0.1858	0.31	0.77	0.4500
**R PLIC**	−0.48	−1.20	0.2426	−0.23	−0.58	0.5671	0.54	1.35	0.1883	0.44	1.10	0.2818
**L PTR**	−0.38	−0.96	0.3451	−0.49	−1.22	0.2342	0.42	1.05	0.3038	0.23	0.58	0.5678
**L RLIC**	−0.38	−0.95	0.3515	−0.39	−0.99	0.3340	0.41	1.03	0.3124	0.77	1.91	0.0672
**R SCR**	−0.34	−0.86	0.4002	−0.38	−0.95	0.3520	0.37	0.93	0.3634	0.31	0.79	0.4389
**L SFO**	−0.31	−0.77	0.4487	−0.35	−0.87	0.3907	0.22	0.56	0.5793	0.05	0.13	0.8959
**R UNC**	−0.31	−0.78	0.4400	−0.38	−0.96	0.3487	0.35	0.87	0.3918	0.22	0.55	0.5860
**L ACR**	−0.30	−0.76	0.4563	−0.34	−0.86	0.3984	0.31	0.78	0.4453	0.28	0.69	0.4950
**L CGC**	−0.29	−0.72	0.4758	−0.20	−0.49	0.6276	0.38	0.95	0.3495	0.45	1.12	0.2737
**L PLIC**	−0.29	−0.74	0.4683	−0.07	−0.17	0.8630	0.38	0.96	0.3486	0.38	0.96	0.3472
**R SFO**	−0.25	−0.62	0.5401	−0.32	−0.80	0.4288	0.30	0.76	0.4557	0.16	0.39	0.6964
**R IFO**	−0.22	−0.55	0.5841	−0.18	−0.45	0.6572	0.16	0.39	0.6996	−0.20	−0.49	0.6299
**L UNC**	−0.22	−0.55	0.5854	−0.29	−0.73	0.4693	0.22	0.54	0.5915	0.07	0.17	0.8639
**R CST**	−0.16	−0.39	0.6985	−0.29	−0.73	0.4712	0.12	0.3	0.7646	−0.79	−1.97	0.0601
**L IFO**	−0.15	−0.39	0.7017	−0.13	−0.32	0.7541	0.19	0.48	0.6321	0.03	0.07	0.9444
**L CST**	−0.14	−0.35	0.7301	−0.29	−0.71	0.4814	−0.13	−0.32	0.7522	−0.80	−2.00	0.0565
**R PCR**	−0.14	−0.35	0.7295	−0.12	−0.30	0.7651	0.28	0.70	0.4931	0.27	0.67	0.5116
**L FXST**	−0.13	−0.32	0.7531	0.01	0.03	0.9769	0.10	0.25	0.8058	0.97	2.43	0.0225
**FX**	−0.09	−0.21	0.8320	0.44	1.09	0.284	0.82	2.04	0.0516	−0.32	−0.80	0.4323
**CC**	−0.08	−0.20	0.8408	0.56	1.41	0.1711	0.44	1.11	0.2770	−0.23	−0.58	0.5682
**SCC**	−0.05	−0.13	0.8943	0.49	1.23	0.2293	0.47	1.17	0.2521	−0.02	−0.04	0.9664
**R PTR**	−0.04	−0.11	0.9139	−0.02	−0.05	0.9602	0.08	0.19	0.8511	0.15	0.37	0.7179
**L EC**	0.03	0.08	0.9365	0.10	0.25	0.8072	−0.07	−0.18	0.8614	0.60	1.51	0.1434
**Average**	0.05	0.13	0.8951	0.17	0.42	0.6815	0.11	0.28	0.7802	0.47	1.18	0.2498
**L PCR**	0.05	0.12	0.9059	0.02	0.06	0.9555	−0.03	−0.07	0.9416	0.43	1.09	0.2882
**R FXST**	0.06	0.14	0.8876	0.27	0.67	0.5074	0.01	0.03	0.9768	0.10	0.25	0.8051
**L SS**	0.11	0.26	0.7936	0.13	0.31	0.7566	−0.10	−0.26	0.7964	0.28	0.69	0.4959
**BCC**	0.20	0.51	0.6156	0.9	2.24	0.0344	0.04	0.10	0.9243	−0.45	−1.13	0.2699
**R SS**	0.25	0.63	0.5343	0.31	0.77	0.4474	−0.30	−0.74	0.4659	0.09	0.21	0.8319
**R EC**	0.38	0.94	0.3560	0.53	1.31	0.2010	−0.50	−1.25	0.2219	0.34	0.85	0.4031
**L SLF**	0.40	1.00	0.3254	0.51	1.27	0.2173	−0.37	−0.91	0.3696	0.37	0.94	0.3584
**R SLF**	0.43	1.07	0.2956	0.35	0.87	0.3902	−0.44	−1.09	0.2872	0.31	0.77	0.4487

Notes: Results from regression model that investigates the effect of total SANS on ROIs after adjusting for age, sex and average motion. The ROIs are ordered by ascending effect sizes for FA_t. ROIs that pass FDR threshold, $p \leq 0.0109$, are indicated in bold.. *Abbreviations:* ACR: anterior corona radiata, AD: axial diffusivity, ALIC: anterior limb of internal capsule, BCC: body of corpus callosum, CC: corpus callosum, CGC: cingulum, CGH: cingulum hippocampal portion, CR: corona radiata, CST: corticospinal tract, EC: external capsule, FA: fractional anisotropy, FX: fornix, FXST: fornix stria terminalis, GCC: genu of corpus callosum, IC: internal capsule, IFO: inferior fronto occipital fasciculus, MD: mean diffusivity, PCR: posterior corona radiata, PLIC: posterior limb of internal capsule, PTR: posterior thalamic radiation, RD: radial diffusivity, RLIC: retrolenticular part of IC, ROI: region of interest, SCC: splenium of corpus callosum, SCR: superior corona radiata, SFO: superior fronto-occipital fasciculus, SLF: superior longitudinal fasciculus, SS: sagittal stratum, UNC: uncinate.

Table S6: Interaction effects between patient status and subcortical structures on the left ACR.

	Dependent: Left ACR FA_t						Dependent: Left ACR AD_t						Dependent: Left ACR RD_t						Dependent: Left ACR Free-water					
	Diagnosis			Diagnosis-by-subcortical structure			Diagnosis			Diagnosis-by-subcortical structure			Diagnosis			Diagnosis-by-subcortical structure			Diagnosis			Diagnosis-by-subcortical structure		
**Subcortical structure**	**d**	**t**	**p**	**d**	**t**	**p**	**d**	**t**	**p**	**d**	**t**	**p**	**d**	**t**	**p**	**d**	**t**	**p**	**d**	**t**	**p**	**d**	**t**	**p**
**L Accumbens**	−0.73	−2.91	**0.0049**	0.25	1.02	0.3124	−0.76	−3.03	**0.0035**	0.19	0.77	0.4453	0.73	2.88	**0.0053**	−0.2	−0.8	0.4268	0.29	1.16	0.2517	−0.19	−0.78	0.4367
**L Amygdala**	−0.72	−2.83	**0.0063**	0.41	1.63	0.1074	−0.72	−2.83	**0.0062**	0.4	1.59	0.1157	0.68	2.67	**0.0096**	−0.39	−1.58	0.12	0.33	1.31	0.1955	−0.48	−1.92	0.0591
**L Caudate**	−0.77	−3.05	**0.0034**	0.29	1.15	0.2536	−0.78	−3.08	**0.003**	0.3	1.19	0.2372	0.74	2.93	**0.0046**	−0.3	−1.22	0.226	0.43	1.7	0.0948	−0.69	−2.79	**0.0069**
**L Hippocampus**	−0.69	−2.75	**0.0077**	0.77	3.11	**0.0028**	−0.7	−2.8	**0.0068**	0.72	2.92	**0.0048**	0.66	2.63	**0.0108**	−0.7	−2.82	**0.0064**	0.29	1.17	0.2456	−0.36	−1.44	0.1539
**L Pallidum**	−0.74	−2.93	**0.0047**	0.4	1.62	0.1098	−0.74	−2.93	**0.0046**	0.38	1.53	0.1306	0.7	2.79	**0.0069**	−0.37	−1.49	0.1414	0.4	1.59	0.1156	−0.15	−0.62	0.5361
**L Putamen**	−0.84	−3.31	**0.0015**	0.62	2.47	0.0161	−0.84	−3.3	**0.0016**	0.6	2.41	0.0188	0.8	3.14	**0.0025**	−0.55	−2.2	0.0312	0.49	1.94	0.0573	−0.17	−0.66	0.5095
**L Thalamus**	−0.85	−3.34	**0.0014**	0.5	2.02	0.0481	−0.86	−3.39	**0.0012**	0.42	1.69	0.0954	0.82	3.24	**0.0019**	−0.43	−1.7	0.0938	0.43	1.71	0.0917	−0.69	−2.76	**0.0075**
**L Ventricle**	−0.73	−2.9	**0.005**	−0.44	−1.76	0.0831	−0.75	−3	**0.0038**	−0.37	−1.5	0.1388	0.72	2.86	**0.0057**	0.34	1.37	0.1754	0.3	1.19	0.2374	−0.43	−1.75	0.085
**R Accumbens**	−0.65	−2.57	0.0124	0.11	0.44	0.6649	−0.68	−2.68	**0.0092**	0.02	0.09	0.9251	0.63	2.5	0.015	−0.06	−0.24	0.8107	0.24	0.94	0.3522	−0.39	−1.56	0.1233
**R Amygdala**	−0.75	−2.98	**0.004**	0.67	2.72	**0.0084**	−0.76	−3.01	**0.0037**	0.63	2.53	0.0139	0.71	2.84	**0.006**	−0.61	−2.45	0.017	0.32	1.28	0.2062	−0.41	−1.65	0.1047
**R Caudate**	−0.78	−3.09	**0.003**	0.41	1.66	0.1026	−0.79	−3.13	**0.0027**	0.43	1.72	0.0908	0.75	2.97	**0.0042**	−0.43	−1.7	0.0934	0.42	1.64	0.1064	−0.71	−2.86	**0.0057**
**R Hippocampus**	−0.66	−2.64	**0.0104**	0.69	2.79	**0.0068**	−0.68	−2.7	**0.0088**	0.61	2.46	0.0165	0.64	2.54	**0.0135**	−0.61	−2.45	0.0172	0.28	1.1	0.2747	−0.37	−1.48	0.1428
**R Pallidum**	−0.77	−3.07	**0.0031**	0.31	1.27	0.2095	−0.77	−3.07	**0.0032**	0.27	1.1	0.2743	0.73	2.92	**0.0048**	−0.26	−1.03	0.3073	0.42	1.66	0.1012	−0.41	−1.66	0.1023
**R Putamen**	−0.93	−3.65	**5e−04**	0.61	2.44	0.0175	−0.92	−3.63	**6e−04**	0.57	2.27	0.0263	0.87	3.45	**0.001**	−0.52	−2.1	0.0398	0.53	2.08	0.0414	−0.38	−1.51	0.1364
**R Thalamus**	−0.8	−3.18	**0.0022**	0.68	2.75	**0.0076**	−0.82	−3.25	**0.0018**	0.67	2.72	**0.0084**	0.77	3.06	**0.0032**	−0.66	−2.66	**0.0098**	0.34	1.33	0.1873	−0.43	−1.73	0.0891
**R Ventricle**	−0.75	−2.98	**0.0041**	−0.41	−1.64	0.1059	−0.78	−3.12	**0.0027**	−0.39	−1.58	0.118	0.75	2.97	**0.0042**	0.39	1.58	0.1188	0.24	0.95	0.344	−0.29	−1.16	0.2506

Notes: Results of *Model* for the effect of interaction between patient and subcortical structures on the left ACR. Structures passing FDR threshold $p \leq 0.0116$are indicated in bold. *Abbreviations*: AD_t: FW adjusted axial diffusivity, ACR: anterior corona radiata, FA_t: FW adjusted fractional anisotropy, FW: free-water, L: Left, RD_t: FW adjusted radial diffusivity, R: Right.

Table S7: Interaction effects between patient status and subcortical structures on the right ACR.

	**Dependent: Right ACR FA_t**						**Dependent: Right ACR AD_t**						**Dependent: Right ACR RD_t**						**Dependent: Right ACR Free-water**					
	**Diagnosis**			**Diagnosis-by-subcortical structure**			**Diagnosis**			**Diagnosis-by-subcortical structure**			**Diagnosis**			**Diagnosis-by-subcortical structure**			**Diagnosis**			**Diagnosis-by-subcortical structure**		
**Subcortical structure**	**d**	**t**	**p**	**d**	**t**	**p**	**d**	**t**	**p**	**d**	**t**	**p**	**d**	**t**	**p**	**d**	**t**	**p**	**d**	**t**	**p**	**d**	**t**	**p**
**L Accumbens**	−0.86	−3.42	**0.0011**	0.2	0.79	0.4305	−0.84	−3.35	**0.0013**	0.1	0.41	0.6836	0.85	3.36	**0.0013**	−0.1	−0.41	0.6825	0.35	1.39	0.1699	−0.11	−0.46	0.6497
**L Amygdala**	−1.01	−3.97	**2e−04**	0.19	0.76	0.4489	−0.97	−3.82	**3e−04**	0.13	0.5	0.6169	0.97	3.83	**3e−04**	−0.14	−0.56	0.5789	0.43	1.7	0.0943	−0.54	−2.16	0.0343
**L Caudate**	−1.02	−4.04	**1e−04**	0.45	1.81	0.0753	−0.99	−3.93	**2e−04**	0.43	1.73	0.0891	0.99	3.94	**2e−04**	−0.45	−1.83	0.0723	0.48	1.91	0.06	−0.67	−2.69	**0.0091**
**L Hippocampus**	−0.92	−3.64	**5e−04**	0.42	1.68	0.0975	−0.89	−3.54	**8e−04**	0.32	1.28	0.2034	0.89	3.54	**7e−04**	−0.32	−1.3	0.1969	0.39	1.55	0.1263	−0.36	−1.43	0.1568
**L Pallidum**	−1	−3.98	**2e−04**	0.4	1.62	0.1104	−0.96	−3.81	**3e−04**	0.39	1.55	0.1252	0.96	3.82	**3e−04**	−0.39	−1.58	0.1196	0.46	1.81	0.0743	−0.23	−0.91	0.3644
**L Putamen**	−1.14	−4.49	**0**	0.25	0.99	0.3254	−1.07	−4.23	**1e−04**	0.19	0.76	0.4514	1.08	4.26	**1e−04**	−0.18	−0.72	0.4729	0.59	2.33	0.0229	−0.12	−0.47	0.6421
**L Thalamus**	−1.06	−4.18	**1e−04**	0.51	2.05	0.0443	−1.02	−4.02	**2e−04**	0.47	1.88	0.0649	1.03	4.05	**1e−04**	−0.48	−1.9	0.0619	0.54	2.14	0.036	−0.56	−2.25	0.0278
**L Ventricle**	−0.95	−3.79	**3e−04**	−0.2	−0.81	0.4193	−0.95	−3.78	**3e−04**	−0.17	−0.68	0.4982	0.95	3.78	**3e−04**	0.16	0.64	0.5273	0.36	1.43	0.1569	−0.35	−1.41	0.1638
**R Accumbens**	−0.82	−3.25	**0.0018**	0.09	0.35	0.7296	−0.8	−3.17	**0.0023**	0	−0.02	0.9845	0.8	3.17	**0.0023**	−0.01	−0.04	0.9711	0.33	1.31	0.1947	−0.47	−1.88	0.0644
**R Amygdala**	−0.96	−3.81	**3e−04**	0.49	1.96	0.0537	−0.92	−3.67	**5e−04**	0.41	1.67	0.1005	0.93	3.68	**5e−04**	−0.42	−1.68	0.0983	0.4	1.6	0.1144	−0.45	−1.82	0.0736
**R Caudate**	−1.01	−3.99	**2e−04**	0.39	1.55	0.1257	−0.99	−3.9	**2e−04**	0.37	1.5	0.1387	0.99	3.91	**2e−04**	−0.4	−1.61	0.1113	0.48	1.91	0.0603	−0.85	−3.39	**0.0012**
**R Hippocampus**	−0.88	−3.49	**9e−04**	0.53	2.14	0.0361	−0.85	−3.39	**0.0012**	0.43	1.72	0.0893	0.85	3.39	**0.0012**	−0.44	−1.76	0.0824	0.37	1.46	0.1482	−0.44	−1.76	0.0835
**R Pallidum**	−1.04	−4.14	**1e−04**	0.28	1.14	0.2571	−1	−3.96	**2e−04**	0.24	0.98	0.3301	1	3.97	**2e−04**	−0.26	−1.03	0.3068	0.48	1.92	0.0587	−0.5	−2.02	0.047
**R Putamen**	−1.14	−4.51	**0**	0.18	0.72	0.472	−1.08	−4.25	**1e−04**	0.12	0.47	0.6428	1.08	4.26	**1e−04**	−0.11	−0.45	0.6541	0.62	2.46	0.0166	−0.28	−1.12	0.2667
**R Thalamus**	−1	−3.98	**2e−04**	0.51	2.04	0.0458	−0.98	−3.89	**2e−04**	0.5	2.03	0.046	0.98	3.9	**2e−04**	−0.51	−2.07	0.0427	0.42	1.65	0.1029	−0.55	−2.21	0.0309
**R Ventricle**	−0.94	−3.72	**4e−04**	−0.03	−0.14	0.8905	−0.93	−3.71	**4e−04**	−0.03	−0.13	0.8958	0.93	3.71	**4e−04**	0.04	0.14	0.8881	0.3	1.2	0.2361	−0.17	−0.69	0.4929

Notes: Results of *Model 2* for the effect of interaction between patient and subcortical structures on the right ACR. Structures passing FDR threshold $p \leq 0.0116$ are indicated in bold. *Abbreviations*: AD_t: FW adjusted axial diffusivity, ACR: anterior corona radiata, FA_t: FW adjusted fractional anisotropy, FW: free-water, L: Left, RD_t: FW adjusted radial diffusivity, R: Right.

Table S8: Interaction effects between patient status and subcortical structures on the left ALIC.

	**Dependent: Left ALIC FA_t**						**Dependent: Left ALIC AD_t**						**Dependent: Left ALIC RD_t**						**Dependent: Left ALIC Free-water**					
	**Diagnosis**			**Diagnosis-by-subcortical structure**			**Diagnosis**			**Diagnosis-by-subcortical structure**			**Diagnosis**			**Diagnosis-by-subcortical structure**			**Diagnosis**			**Diagnosis-by-subcortical structure**		
**Subcortical structure**	**d**	**t**	**p**	**d**	**t**	**p**	**d**	**t**	**p**	**d**	**t**	**p**	**d**	**t**	**p**	**d**	**t**	**p**	**d**	**t**	**p**	**d**	**t**	**p**
**L Accumbens**	−0.7	−2.8	**0.0068**	−0.14	−0.56	0.5742	−0.73	−2.91	**0.0049**	−0.1	−0.4	0.6877	0.71	2.83	**0.0062**	0.13	0.51	0.6092	−0.39	−1.55	0.1262	0.07	0.28	0.7828
**L Amygdala**	−0.66	−2.61	**0.0113**	0.13	0.51	0.6147	−0.66	−2.62	**0.0109**	0.19	0.75	0.4552	0.66	2.6	**0.0116**	−0.08	−0.32	0.7529	−0.31	−1.21	0.2315	−0.15	−0.59	0.5596
**L Caudate**	−0.77	−3.04	**0.0034**	0.19	0.78	0.441	−0.8	−3.2	**0.0021**	0.3	1.19	0.2373	0.77	3.06	**0.0032**	−0.25	−0.99	0.3263	−0.36	−1.45	0.1527	−0.56	−2.28	0.0262
**L Hippocampus**	−0.68	−2.69	**0.0091**	0.18	0.71	0.4778	−0.7	−2.77	**0.0072**	0.2	0.82	0.4179	0.69	2.73	**0.0082**	−0.18	−0.72	0.4744	−0.36	−1.41	0.1624	−0.16	−0.65	0.5162
**L Pallidum**	−0.7	−2.79	**0.0069**	0.09	0.38	0.7085	−0.7	−2.78	**0.0071**	0.12	0.49	0.6232	0.69	2.75	**0.0077**	−0.09	−0.36	0.7186	−0.33	−1.33	0.1882	−0.19	−0.76	0.4497
**L Putamen**	−0.61	−2.42	**0.0183**	0.36	1.46	0.1495	−0.63	−2.49	0.0153	0.44	1.77	0.0808	0.62	2.45	0.0169	−0.34	−1.35	0.1823	−0.25	−0.99	0.3276	0.07	0.29	0.7691
**L Thalamus**	−0.67	−2.65	**0.0102**	0.09	0.36	0.7173	−0.69	−2.71	**0.0087**	0.12	0.48	0.6338	0.69	2.71	**0.0087**	−0.1	−0.42	0.6765	−0.29	−1.15	0.2557	−0.16	−0.65	0.5182
**L Ventricle**	−0.87	−3.44	**0.001**	−0.11	−0.43	0.6718	−0.91	−3.62	**6e−04**	−0.06	−0.24	0.8098	0.93	3.71	**4e−04**	0.12	0.5	0.622	−0.43	−1.72	0.0896	−0.28	−1.14	0.2605
**R Accumbens**	−0.69	−2.74	**0.008**	0.12	0.48	0.6326	−0.73	−2.89	**0.0053**	0.18	0.71	0.4822	0.68	2.69	**0.009**	−0.09	−0.37	0.7141	−0.44	−1.75	0.0856	0.07	0.28	0.7838
**R Amygdala**	−0.68	−2.72	**0.0083**	0.11	0.45	0.6512	−0.72	−2.85	**0.0058**	0.15	0.61	0.5454	0.69	2.75	**0.0076**	−0.08	−0.33	0.7426	−0.38	−1.49	0.1406	−0.02	−0.08	0.9348
**R Caudate**	−0.75	−2.95	**0.0045**	0.07	0.28	0.7821	−0.78	−3.08	**0.0031**	0.14	0.56	0.5757	0.76	2.99	**0.0039**	−0.11	−0.43	0.6686	−0.37	−1.47	0.1456	−0.65	−2.62	**0.0111**
**R Hippocampus**	−0.66	−2.63	**0.0108**	0.22	0.91	0.3678	−0.7	−2.77	**0.0073**	0.21	0.83	0.408	0.68	2.69	**0.0091**	−0.24	−0.97	0.3352	−0.36	−1.44	0.1541	−0.14	−0.56	0.576
**R Pallidum**	−0.71	−2.84	**0.0061**	0.01	0.06	0.9562	−0.72	−2.86	**0.0056**	0.06	0.23	0.8174	0.71	2.82	**0.0064**	−0.01	−0.04	0.9697	−0.32	−1.27	0.2101	−0.25	−1.01	0.3146
**R Putamen**	−0.68	−2.67	**0.0096**	0.3	1.19	0.2375	−0.7	−2.76	**0.0075**	0.39	1.56	0.1246	0.68	2.7	**0.009**	−0.28	−1.11	0.2696	−0.28	−1.11	0.2705	−0.16	−0.64	0.5251
**R Thalamus**	−0.68	−2.71	**0.0087**	0.01	0.03	0.9736	−0.7	−2.78	**0.0072**	0	0	0.9985	0.69	2.75	**0.0078**	−0.01	−0.03	0.977	−0.36	−1.44	0.1551	−0.17	−0.68	0.497
**R Ventricle**	−0.81	−3.21	**0.0021**	−0.31	−1.26	0.2119	−0.84	−3.33	**0.0014**	−0.25	−0.99	0.3247	0.85	3.38	**0.0012**	0.35	1.41	0.1632	−0.44	−1.75	0.0851	−0.35	−1.39	0.1687

Notes: Results of *Model 2* for the effect of interaction between patient and subcortical structures on the left ALIC. Structures passing FDR threshold $p \leq 0.0116$ are indicated in bold.
Abbreviations: AD_t: FW adjusted axial diffusivity, ALIC: anterior limb of internal capsule, FA_t: FW adjusted fractional anisotropy, FW: free-water, L: Left, RD_t: FW adjusted radial diffusivity, R: Right.

Table S9: Interaction effects between patient status and subcortical structures on the fornix.

	**Dependent: Fornix FA_t**						**Dependent: Fornix AD_t**						**Dependent: Fornix RD_t**						**Dependent: Fornix Free-water**					
	**Diagnosis**			**Diagnosis-by-subcortical structure**			**Diagnosis**			**Diagnosis-by-subcortical structure**			**Diagnosis**			**Diagnosis-by-subcortical structure**			**Diagnosis**			**Diagnosis-by-subcortical structure**		
**Subcortical structure**	**d**	**t**	**p**	**d**	**t**	**p**	**d**	**t**	**p**	**d**	**t**	**p**	**d**	**t**	**p**	**d**	**t**	**p**	**d**	**t**	**p**	**d**	**t**	**p**
**L Accumbens**	−0.15	−0.61	0.5419	0.19	0.78	0.4385	0.25	1.01	0.3179	0.05	0.21	0.8337	0.76	3.04	**0.0034**	−0.73	−2.95	**0.0044**	0.18	0.73	0.4651	0.04	0.16	0.877
**L Amygdala**	−0.27	−1.05	0.2961	0.05	0.22	0.8295	0.22	0.85	0.3983	0.02	0.08	0.9335	0.86	3.41	**0.0011**	−0.47	−1.88	0.0644	0.28	1.09	0.2808	0.11	0.44	0.6631
**L Caudate**	−0.22	−0.89	0.3765	−0.07	−0.3	0.7674	0.21	0.84	0.4019	0.01	0.04	0.9672	0.81	3.23	**0.0019**	0	0	0.9998	0.22	0.89	0.378	0.02	0.09	0.9296
**L Hippocampus**	−0.21	−0.82	0.4124	0.08	0.33	0.7415	0.23	0.91	0.3678	0	0.02	0.9856	0.83	3.29	**0.0016**	−0.68	−2.72	**0.0083**	0.21	0.85	0.3991	0.13	0.51	0.6117
**L Pallidum**	−0.25	−0.98	0.3293	0.03	0.1	0.9175	0.19	0.76	0.452	0.01	0.05	0.9618	0.83	3.3	**0.0016**	−0.46	−1.85	0.0689	0.28	1.09	0.2776	0.19	0.78	0.4385
**L Putamen**	−0.34	−1.35	0.1821	−0.08	−0.31	0.7561	0.15	0.61	0.5465	0.02	0.08	0.9374	0.94	3.71	**4e−04**	−0.48	−1.9	0.0615	0.34	1.33	0.1892	0.32	1.27	0.2093
**L Thalamus**	−0.26	−1.03	0.3061	0.2	0.79	0.4321	0.22	0.87	0.3873	0.17	0.68	0.497	0.93	3.67	**5e−04**	−0.6	−2.4	0.0192	0.25	0.99	0.3253	−0.05	−0.2	0.8387
**L Ventricle**	−0.05	−0.21	0.8342	0.03	0.12	0.9061	0.44	1.75	0.0843	−0.2	−0.79	0.4332	0.8	3.17	**0.0023**	0.32	1.3	0.197	0.03	0.14	0.8898	−0.45	−1.8	0.0767
**R Accumbens**	−0.23	−0.9	0.3706	0.08	0.31	0.7563	0.2	0.81	0.4232	0.02	0.09	0.926	0.72	2.83	**0.0062**	−0.43	−1.71	0.0913	0.29	1.15	0.2549	0.01	0.05	0.9587
**R Amygdala**	−0.22	−0.87	0.3862	0.15	0.59	0.5557	0.24	0.95	0.347	0.11	0.45	0.6527	0.88	3.5	**8e−04**	−0.54	−2.18	0.0331	0.21	0.83	0.4076	−0.09	−0.35	0.7277
**R Caudate**	−0.23	−0.9	0.3712	0.06	0.23	0.8189	0.21	0.83	0.412	0.17	0.68	0.4993	0.82	3.25	**0.0019**	−0.11	−0.43	0.6665	0.23	0.89	0.3763	−0.07	−0.28	0.7784
**R Hippocampus**	−0.16	−0.63	0.5291	0.19	0.78	0.4393	0.28	1.09	0.2777	0.13	0.52	0.6053	0.83	3.28	**0.0017**	−0.66	−2.65	**0.01**	0.16	0.64	0.5239	−0.04	−0.15	0.8797
**R Pallidum**	−0.22	−0.89	0.3771	0.08	0.34	0.7357	0.22	0.87	0.3864	0.02	0.09	0.9307	0.86	3.43	**0.0011**	−0.5	−2.02	0.048	0.23	0.92	0.3601	0.15	0.62	0.5369
**R Putamen**	−0.36	−1.4	0.1655	0.02	0.07	0.9423	0.13	0.51	0.6129	0.13	0.53	0.5947	0.94	3.73	**4e−04**	−0.33	−1.33	0.1889	0.33	1.28	0.2041	0.1	0.39	0.6967
**R Thalamus**	−0.26	−1.04	0.3019	0.33	1.33	0.1872	0.2	0.81	0.4225	0.35	1.41	0.1621	0.83	3.3	**0.0016**	−0.32	−1.29	0.1999	0.25	0.99	0.3237	−0.14	−0.57	0.5678
**R Ventricle**	0	0.01	0.9952	0.42	1.7	0.0948	0.5	2	0.05	−0.01	−0.03	0.9759	0.82	3.24	**0.0019**	0.03	0.11	0.9143	−0.06	−0.26	0.7974	−0.69	−2.78	**0.0071**

Notes: Results of *Model 2* for the effect of interaction between patient and subcortical structures on the fornix. Structures passing FDR threshold $p \leq 0.0116$ are indicated in bold.
Abbreviations: AD_t: FW adjusted axial diffusivity, FA_t: FW adjusted fractional anisotropy, FW: free-water, L: Left, RD_t: FW adjusted radial diffusivity, R: Right.
